# Deficiency of FABP7 Triggers Premature Neural Differentiation in Idiopathic Normocephalic Autism Organoids

**DOI:** 10.1002/advs.202406849

**Published:** 2024-11-18

**Authors:** Xiao Han, Yuanlin He, Yuanhao Wang, Wenzhu Hu, Chu Chu, Lei Huang, Yuan Hong, Lu Han, Xu Zhang, Yao Gao, Yuan Lin, Hongxia Ma, Hongbing Shen, Xiaoyan Ke, Yan Liu, Zhibin Hu

**Affiliations:** ^1^ Interdisciplinary Inno Center for Organoids, State Key Laboratory of Reproductive Medicine and Offspring Health Nanjing Medical University Nanjing 211166 China; ^2^ Institute of Stem Cell and Neural Regeneration, School of Pharmacy Nanjing Medical University Nanjing 211166 China; ^3^ State Key Laboratory of Reproductive Medicine (Suzhou Centre), The Affiliated Suzhou Hospital of Nanjing Medical University, Suzhou Municipal Hospital, Gusu School, Innovation Center of Suzhou Nanjing Medical University Suzhou 215000 China; ^4^ Department of Epidemiology and Biostatistics, Center for Global Health, School of Public Health Nanjing Medical University Nanjing 211166 China; ^5^ Autism Research Center, State Key Laboratory of Reproductive Medicine The Affiliated Brain Hospital of Nanjing Medical University Nanjing 210029 China; ^6^ Department of Maternal, Child and Adolescent Health, School of Public Health Nanjing Medical University Nanjing 211166 China

**Keywords:** autism spectrum disorder, cerebral organoid, FABP7, normocephaly, premature neural differentiation

## Abstract

Autism spectrum disorder (ASD), which is caused by heterogeneous genetic and environmental factors, is characterized by diverse clinical phenotypes linked to distinct pathological mechanisms. ASD individuals with a shared clinical phenotype might contribute to revealing the molecular mechanism underlying ASD progression. Here, it is generated induced pluripotent stem cell (iPSC)‐derived cerebral organoids from normocephalic individuals with ASD in a prospective birth cohort with a shared clinical diagnosis. Multiple cell lines and time series scRNA‐seq combined with a histomorphological analysis revealed premature neural differentiation of neural stem cells (NSCs) and decreased expression of Fatty acid binding protein 7 (FABP7) in ASD organoids. It is subsequently revealed alterations in the phosphorylation levels of Mitogen‐Activated Protein Kinase Kinase 1/2 (MEK1/2), which are downstream of FABP7, and the regulation of the FABP7/MEK pathway reversed improper neural differentiation in the ASD organoids. Moreover, both Fabp7‐knockdown and MEK2‐overexpressing mice exhibited repetitive stereotyped behaviors and social defects relevant to autism. This study reveals the role of the FABP7/MEK pathway in abnormal NSC differentiation in normocephalic individuals with ASD, which might provide a promising therapeutic target for ASD treatment.

## Graphical abstract

The iPSC‐derived cerebral organoids are generated from normocephalic individuals with ASD. Subsequent analyses, including multiple cell lines and time‐series scRNA‐seq combined with histologic analysis, revealed premature neural differentiation of neural stem cells. Notably, modulation of the FABP7/MEK pathway can reverse aberrant neural differentiation in the ASD organoids. Moreover, both *Fabp7*‐knockdown and *MEK2*‐overexpressing mice exhibited social behaviors indicative of autism.

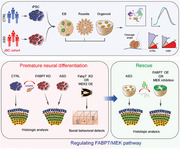

## 1 Introduction

Autism spectrum disorder (ASD) is a neurodevelopmental disorder associated with intellectual impairment in children and is characterized by social and communication deficits accompanied by stereotyped, repetitive behaviors. Given that the heterogeneity of ASD might hinder the elucidation of pathological mechanisms, focusing on specific clinical phenotypes could reveal the potential pathopoiesis mechanism of ASD. An increasing number of studies have reported that individuals with idiopathic ASD can be divided into distinct patterns based on the head circumference or onset status, including macrocephaly and normocephaly^[^ ^1^, ^2^ ^]^ or early onset^[^ ^3^, ^4^ ^]^ and regression.^[^ ^5^, ^6^ ^]^ Inherited variants and/or rare de novo variants^[^ ^7^, ^8^, ^9^ ^]^ have been reported to be involved in the etiology of some subtypes of ASD. Thus, the heterogeneity of genetic and environmental factors that cause distinct phenotypes makes elucidating the molecular mechanisms of ASD a major challenge.

With the advent of induced pluripotent stem cell (iPSC) and brain organoid technologies, patient‐derived neurons or organoids have revealed the convergence of neuronal morphological features and gene expression patterns in individuals with ASD.^[^ ^2^, ^10^, ^11^, ^12^, ^13^ ^]^ However, the average age at ASD diagnosis ranges from 3 to 10 years,^[^ ^14^ ^]^ and the diagnoses include both early‐onset ASD and regressive ASD, resulting in heterogeneity, which makes mechanistic studies difficult. Numerous factors were reported to be involved in the etiology of ASD in previous studies, including studies of patient‐derived iPSCs and animal models.^[^ ^2^, ^15^, ^16^, ^17^, ^18^, ^19^ ^]^ For example, *FOXG1* mediated an imbalance in inhibitory/excitatory signals in macrocephalic ASD telencephalic organoids.^[^ ^2^ ^]^ However, whether risk genes participate in the pathogenesis of ASD is still unclear.

In this study, we identified patients with ASD at the age of 2 years and excluded known genetic variants^[^ ^7^ ^]^ and environmental risk factors,^[^ ^20^, ^21^ ^]^ such as maternal type 1 diabetes, advanced maternal age, maternal gestational hypertension and prepregnancy maternal antidepressant use, in our birth cohort. We then generated iPSCs and cerebral organoids from three individuals with idiopathic nonregressive ASD presenting with normocephaly. Multiple cell lines and time series single‐cell sequencing (scRNA‐seq) revealed premature differentiation of neural stem cells (NSCs) in ASD organoids as well as defective expression of fatty acid binding protein 7 (FABP7), which is a conserved protein that binds to hydrophobic ligands and long‐chain fatty acids. FABP7 is highly expressed in NSCs and astrocytes in the brain^[^ ^22^ ^]^ and is critical for the establishment of radial glial fibers and the maintenance of neuroepithelial cells.^[^ ^23^, ^24^ ^]^ However, the mechanism by which FABP7 regulates neurogenesis and cortical differentiation in the pathogenesis of ASD is still unclear. Therefore, we found that Mitogen‐Activated Protein Kinase Kinase 1/2 (MEK1/2) functions downstream of *FABP7* in regulating the premature differentiation of NSCs. Inhibition of *Fabp7* or forced expression of *MEK2* in the dentate gyrus (DG) of the hippocampus led to autistic‐like behaviors in mice. Our study revealed impaired neural differentiation in NSCs from the ASD group, which might provide novel mechanistic insights into the pathogenesis of ASD.

## 2 Results

### 2.1 Cerebral Organoids from Normocephalic Individuals with ASD Exhibit Premature Neural Differentiation

**Figure 1:**
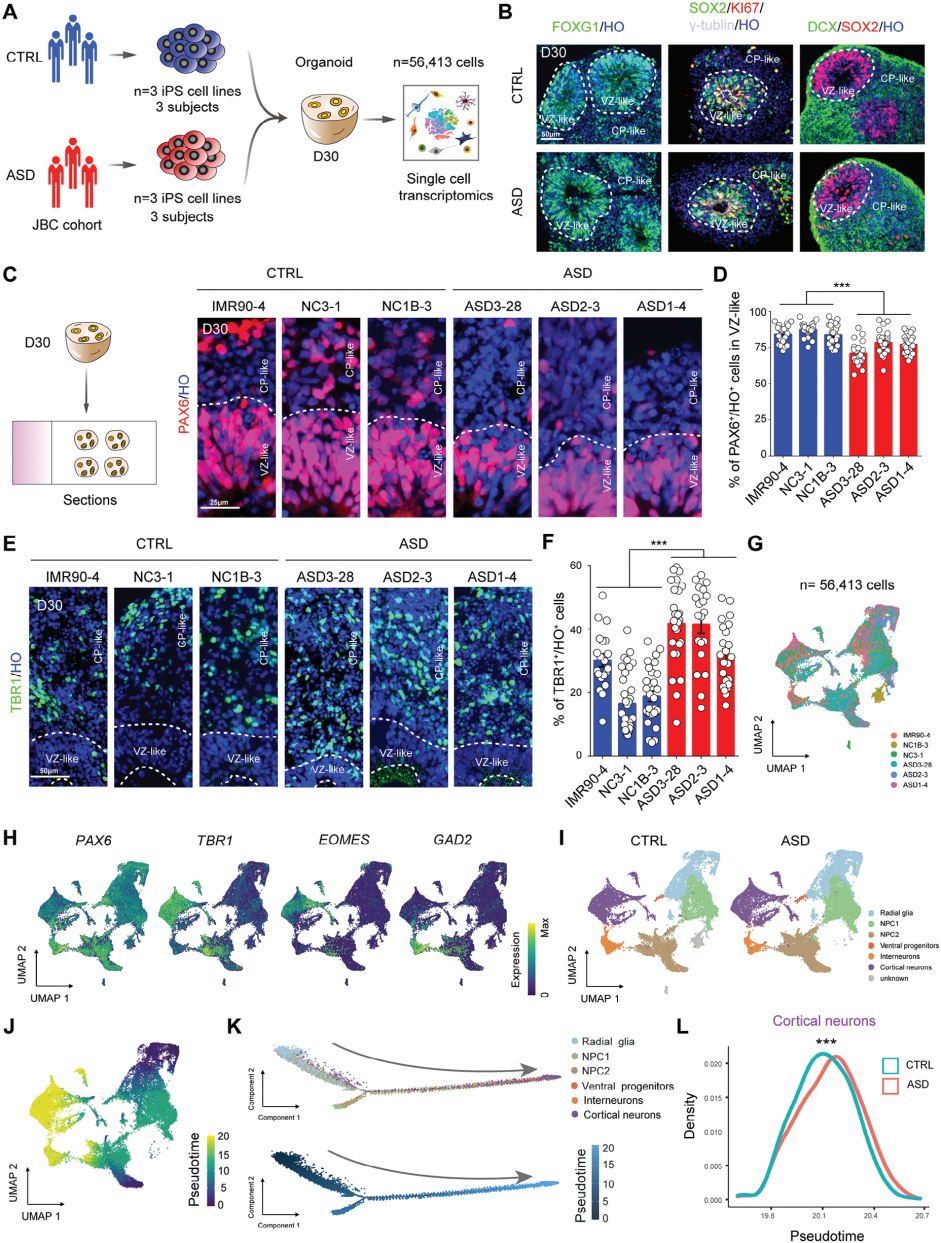
Histomorphological analysis combined with single‐cell RNA‐seq of multiple cell lines reveals premature neural differentiation in normocephalic ASD cerebral organoids. A) Schematic diagram displaying the construction of cerebral organoids derived from normal and ASD individuals (n = 3 control iPS cell lines: IMR90‐4, NC1B‐3 and NC3‐1, n = 3 ASD iPS cell lines: ASD3‐28, ASD2‐3 and ASD1‐4) and scRNA‐seq analysis of cerebral organoids at D30 (total cells: n = 56413 cells). B) Immunofluorescence assays of brain organoids at D30 showing the telencephalic marker FOXG1 (green, left panel), the neural stem cell marker SOX2 (green in middle panel, red in right panel), the proliferation marker KI67 (red, middle panel), the centrosomal and adherens junction marker γ‐tubulin (gray, middle panel) and the newborn neuron marker DCX (green, right panel). CP: cortical plate, HO: Hoechst staining, VZ: ventricular zone. Scale bar: 50 µm. C) Representative images of immunostaining for the neural progenitor marker PAX6 and quantification of the proportion of PAX6^+^/HO^+^ in the VZ‐like zone in the CRTL and ASD D30 organoids. Scale bar: 50 µm. D) Histograms showing the ratio of PAX6^+^/HO^+^ cells in the VZ‐like zone of the CRTL and ASD organoids at D30. (IMR90‐4: n = 20 VZ‐like regions, NC3‐1: n = 26 VZ‐like regions, NC1B‐3: n = 23 VZ‐like regions, ASD3‐28: n = 20 VZ‐like regions, ASD2‐3: n = 28 VZ‐like regions, ASD1‐4: n = 23 VZ‐like regions, ASD versus CTRL: ***p < 0.001). VZ‐like regions from 3 independent biological replicate experiments were analyzed for each cell line. E) Representative images of D30 ASD and control organoids with staining of deep layer marker TBR1 and quantitation of TBR1^+^/HO^+^ cells in the CP‐like zone. Scale bar: 50 µm. F) Histograms displaying the ratio of TBR1^+^/HO^+^ cells in the CRTL and ASD organoids at D30. (IMR90‐4: n = 20 organoids, NC3‐1: n = 32 organoids, NC1B‐3: n = 30 organoids, ASD3‐28: n = 27 organoids, ASD2‐3: n = 20 organoids, ASD1‐4: n = 23 organoids, ASD versus CTRL: ***p < 0.001). Organoids from 3 independent biological replicate experiments were analyzed for each cell line. G) UMAP plots colored on 6 cell lines at D30 (total cells: n = 56413 cells). H) UMAP plot of cell types annotated according to expression of known marker genes in CRTL (n = 3) and ASD (n = 3) cerebral organoids at D30. NPC: neural progenitor cells. I) UMAP plots colored on gene expression of representative genes used to assign cluster identities. The color scale represents normalized expression for each cell for a given gene with the “LogNormalize” method in the R package Seurat 3.2.2. J) UMAP plot of all cells at D30 colored by pseudotime. Cells appearing earliest in pseudotime are denoted by dark purple, and those latest in pseudotime are in yellow. K) Ordering of the entire pooled scRNA‐seq expression data according to the pseudotime position. L) Distributions of cortical neurons in CRTL and ASD organoids over pseudotime. Student's t‐test was used to determine the significance of the average pseuditime of “Cortical neurons” between ASD and CTRL organoids.

We identified 11 toddlers with idiopathic nonregressive ASD and a normal head circumference in the Jiangsu Birth Cohort (JBC),^[^ ^25^ ^]^ 3 of whom were recruited for this study (Figure S1A and Table S1, Supporting Information). We generated iPSCs from three individuals with idiopathic normocephalic ASD (ASD1‐4, ASD2‐3 and ASD3‐28) and used three normal iPSC lines (IMR90‐4, NC3‐1 and NC1B‐3), including age‐ and sex‐matched cell lines, as controls (CTRL) to investigate the etiology of ASD (**Figure** **1**A; **Table** **1**). Immunostaining revealed that the iPSC colonies expressed pluripotent markers (NANOG and SOX2) and a proliferative marker (KI67) (Figure S1B,C, Supporting Information). We induced iPSC differentiation using a modified protocol with dual‐SMAD inhibitors (DMH1 and SB431542) to generate cerebral organoids.^[^ ^26^ ^]^ Staining for FOXG1, SOX2 and DCX revealed that the ASD organoids acquired a telencephalic identity and formed classic layered structures similar to those of the control organoids at Day 30 (D30) (Figure 1B). We first calculated the area of the ventricular zone (VZ)‐like region at D30 to investigate the pathological phenotypes of normocephalic ASD cerebral organoids and found that the area of the VZ‐like region in the ASD organoids was significantly decreased compared with that in the controls (Figure S1D, Supporting Information). Then, we performed immunostaining for PAX6 to determine the number of radial glial cells (RGCs) in D30 organoids and observed that the percentage of PAX6^+^ cells in the VZ‐like region of the ASD organoids was significantly reduced compared to that in the CTRL organoids (Figure 1C,D). Moreover, we measured the number of deep layer neurons using the layer VI marker TBR1 and unexpectedly observed that the number of deep layer neurons was markedly increased in the ASD organoids compared with the CTRL organoids (Figure 1E,F). Our findings indicate that premature cortical development might occur in ASD organoids compared to the control organoids.

**Table 1:** Basic information of iPSC lines used in this study.

Cell line name	Gender	Age	Source
IMR90‐4	Female	16 pcw	CCL‐186
NC3‐1	Female	36 years	Liu lab
NC1B‐3	Male	5 years	Liu lab
Ihtc‐03	Female	28 years	Liu lab
ASD1‐4	Male	28 months	Liu lab
ASD2‐3	Male	25 months	Liu lab
ASD3‐28	Male	25 months	Liu lab
TASD2A‐1	Male	7 years	Liu lab
TASD2B‐1	Male	7 years	Liu lab
TASD3A‐1	Male	3 years	Liu lab

We performed single‐cell RNA sequencing (scRNA‐Seq) of D30 organoids derived from three normocephalic ASD iPSC lines and three CTRL iPSC lines (ASD iPSC lines: ASD1‐4, ASD2‐3, and ASD3‐28; control iPSC lines: IMR90‐4, NC3‐1 and NC1B‐3) to further systematically profile the cerebral organoid transcriptome (**Table** **1**). A total of 56413 (CTRL 21640 versus ASD 34773) cells were profiled utilizing 10x Genomics Chromium, and each sample had a similar cell composition, based on the transcriptome, and formed a relatively consistent cluster after dimensionality reduction (uniform manifold approximation and projection, UMAP) (Figure 1G). Using the VoxHunt algorithm,^[^ ^27^ ^]^ we confirmed that the transcriptomic identity of cells in brain organoids was highly mapped onto the embryonic 13.5 (E13.5) mouse dorsal forebrain (Figure S1E, Supporting Information). We next identified six major cell types according to the expression of cellular lineage‐specific marker genes that were defined in published datasets^[^ ^28^, ^29^ ^]^ (Figure 1H,I; Figure S1F; Tables S2 and S3, Supporting Information). We calculated that the proportions of cells in each cluster, radial glia and NPC1, were similar, ≈20%. A 10% increase in the proportion of NPC2 and a 5% decrease in the proportion of cortical neurons were observed in the ASD group, but obvious individual heterogeneity was noted (Figure S1G, Supporting Information). Additionally, the proportion of cells in the division phase (G2M) was slightly greater than that in the control organoids (Figure S1H,I, Supporting Information). Next, we performed a pseudotime analysis to reconstruct lineage relationships and found that the cell trajectory was similar across all the iPSC lines between the ASD and CTRL groups (Figure 1J,K; Figure S1J,K, and Table S4, Supporting Information). The boxplot of the pseudotime and each cell type further clarified our speculation that the cellular developmental stage was positively correlated with pseudotime (Figure S1I, Supporting Information), whereas a significant change in the developmental state of the terminally differentiated cortical neuron trajectory was detected in the ASD organoids compared with the developmental state of the CTRL organoids (Figure 1I), which corroborated the premature cortical development in the ASD organoids.

### 2.2 Time Series Single‐Cell RNA‐Seq Reveals Successive Premature Differentiation

**Figure 2:**
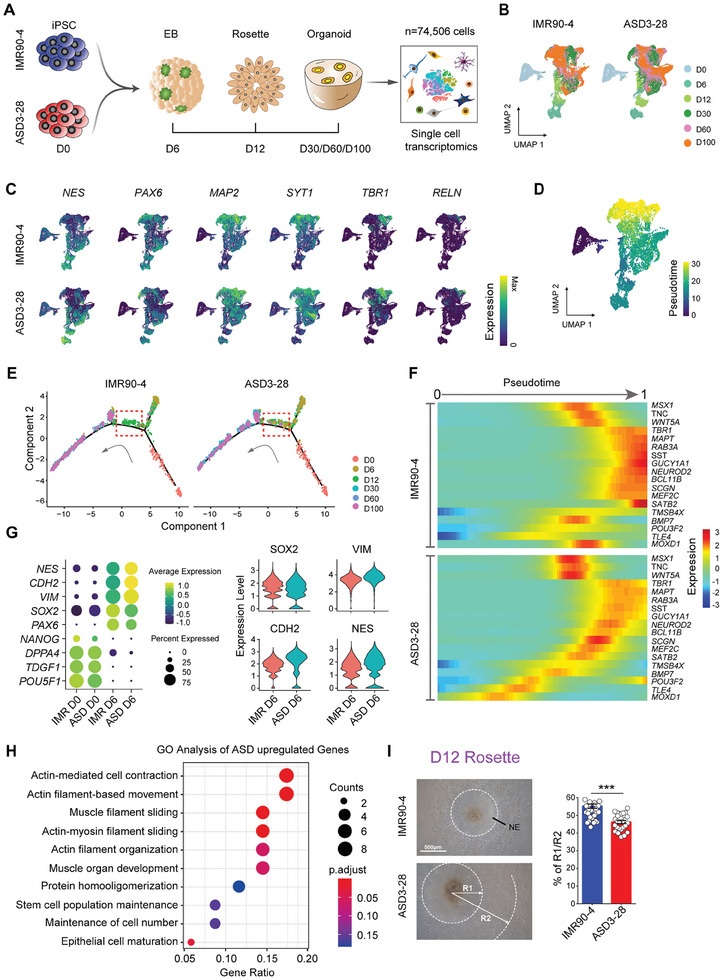
Time series of single‐cell RNA‐seq data illustrating dysplasia of neural epithelial cells associated with premature neural differentiation in ASD organoids. A) Schematic diagram showing the strategies for scRNA‐seq analysis at D0, D6, D12, D30, D60 and D100 in IMR90‐4 and ASD3‐28 cerebral organoids (total cells: n = 74506 cells). B) scRNA‐seq was performed on IMR90‐4 and ASD3‐28 iPSC‐derived cells at different time points during cerebral organoid differentiation from pluripotency, and UMAP plots were generated on the basis of 6 developmental time points. C) UMAP plots colored on gene expression of representative genes used to assign cluster identities between IMR90‐4 and ASD3‐28 during cerebral organoid differentiation from pluripotency, and MAP2, SYT1, TBR1 and RELN showed more distribution in the ASD3‐28 group. D) UMAP reduction of all cells over 6 developmental time points colored by pseudotime. Cells appearing earliest in pseudotime are denoted by dark purple, and those latest in pseudotime are in yellow. E) Ordering of the entire pooled scRNA‐seq expression data according to pseudotime position, and dotted boxes indicate that cells from D6 in the ASD3‐28 group entered the trajectory earlier than those in the IMR90‐4 group. F) Representative genes with significantly differential trajectories over pseudotime in IMR90‐4 and ASD3‐28 cells calculated by Monocle2. G) Dot plots and violin plots showing the expression of pluripotency‐related genes (*POU5F1, NANOG, TDGF1*, and *DPPA4*) and early neural differentiation‐related genes (*NES, CDH2*, and *VIM*) expressed at D6. H) GO analysis of ASD‐enriched genes. Selected GO terms with FDRs of less than 0.05 are shown. I) Optical imaging of IMR90‐4 and ASD3‐28 rosettes at D12 (Left) and histograms (Right) displays the ratio of R1/R2 of IMR90‐4 and ASD3‐28 rosettes (IMR90‐4: n = 35 rosette colonies, ASD3‐28: n = 27 rosette colonies, ***p < 0.001). R: radius, Scale bar: 500 µm. Rosette colonies from 3 independent biological replicate experiments were analyzed for each cell line.

We used IMR90‐4, a standard iPSC line, and ASD3‐28, which was the first ASD iPSC line we generated from an individual with a more severe clinical phenotype (Table S1, Supporting Information), for systematic temporal scRNA‐seq on D0 (iPSCs), D6 (embryoid bodies, EBs), D12 (rosettes), and D30/D60/D100 (organoids) to further characterize the entire process of differentiation from iPSCs to cerebral organoids between the ASD and CTRL groups. A total of 74506 cells at six different time points were profiled (**Figure** **2**A). UMAP revealed that the cells were grouped by their developmental state (Figure 2B). Consistent with the analysis strategy described above and based on the expression of marker genes and lineage‐specific marker expression defined in published datasets^[^ ^28^, ^29^ ^]^ (Figure S2A; Tables S3 and S5, Supporting Information), we identified 13 major cell types and observed differences in the proportions of different cell types after D12 (Figure S2B, Supporting Information). We then calculated the proportion of cells in each cluster at distinct stages and found that the cell proportions continuously differed after D6 (Figure S2B, Supporting Information). Additionally, a feature plot indicated that neural differentiation‐related genes were differentially expressed in the ASD organoids (Figure 2C).

Next, the pseudotime analysis revealed distinct trajectories of iPSC differentiation, which were well matched with differentiation at six time points (Figure 2D). We then clustered significantly different gene trajectories and identified six similar expression patterns (Figure S2C,D and Table S5, Supporting Information). The Gene Ontology (GO) enrichment analysis revealed that genes related to the regulation of neuron projection development, axonogenesis, the glutamate receptor signaling pathway and synapse organization were enriched in Cluster 1 and Cluster 2, whose expression gradually increased with pseudotime (Figure S2E and Table S6, Supporting Information). This result further confirmed the accuracy of our constructed forebrain organoids. Along pseudotime, a branchpoint was discovered at D6, and the red dotted boxes suggest that the cells from D6 in the ASD3‐28 groups entered the trajectory earlier than those in the IMR90‐4 groups did (Figure 2E). We also investigated individual pseudotime‐dependent genes and identified differentially expressed pseudotime‐dependent genes (Figure 2F). The data revealed that genes associated with neuronal differentiation, such as *TBR1, NEUROD2, BCL11B, SATB2* and *POU3F2*, were expressed earlier in the ASD groups than in the IMR90‐4 groups. Given these results, transcriptomic profiling at multiple time points indicates the premature differentiation of cerebral organoids derived from patients with ASD.

### 2.3 Dysplasia of ASD Neural Epithelial Cells is Associated with Premature Differentiation

Next, we explored whether the ASD organoids presented a prematurely differentiated phenotype at the cellular level at D6, D12 and D30 (Figure 2A). The statistical analysis showed that the diameter of EBs and the ratio of SOX2^+^ cells in the ASD group were similar to those in the CTRL group at D6 (Figure S2F,G, Supporting Information). Nevertheless, the transcriptomic analysis revealed that the ratio of SOX2^+^ cells in the ASD group was lower than that in the CTRL group (Figure S2H, Supporting Information). Additionally, the expression of stem cell pluripotency‐related genes (*POU5F1, NANOG, TDGF1*, and *DPPA4*) did not differ at D0, whereas early neural differentiation‐related genes (*NES, CDH2*, and *VIM*) were differentially expressed between the ASD and CTRL groups at D6 (Figure 2G).

At D12, a volcano plot revealed that actin filament‐related genes (*ACTA1* and *ACTA2*) were highly expressed in the ASD group, and the GO analysis revealed that neuroepithelial (NE) cells of ASD origin were enriched for actin filament‐based movement and epithelial cell maturation terms (Figure 2H; Figure S2I and Table S7, Supporting Information). Consistently, we found that the radius of the total colony (R2) and central neuroepithelial zone (R1) in the ASD group were dramatically increased compared with those in the CTRL group (Figure 2I), which demonstrated dysplasia in ASD rosettes, including abnormal cell migration or premature differentiation of ASD NE cells.

Thus, we observed continuous dysplasia of neural stem cells (NSCs) related to the premature differentiation of EBs and rosettes in the ASD group.

### 2.4 An Increased Number of RG Cells Underwent Horizontal Cleavage in Normocephalic ASD Organoids

**Figure 3:**
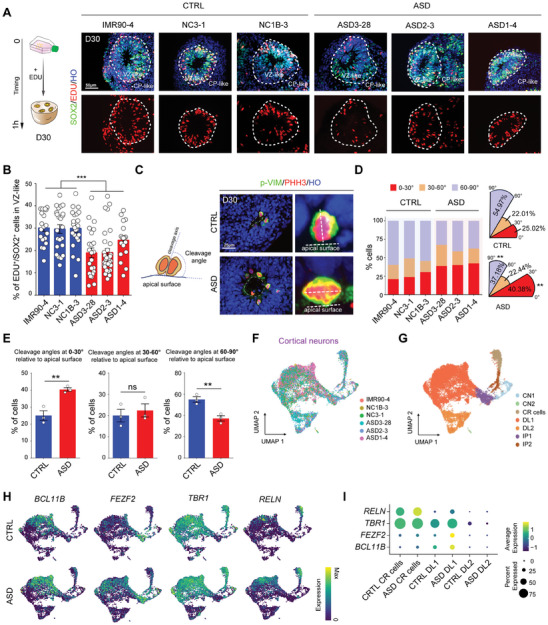
A greater number of RG cells undergoes horizontal cleavage, leading to premature neural differentiation in the ASD organoids. A) Schematic diagram of EdU treatment for 1 h to label proliferating neural progenitors (Left). Representative images of D30 ASD and control organoids stained with SOX2, 1 h EdU and Hoechst (Right). Scale bar: 50 µm. B) Histograms showing the ratio of EdU^+^/SOX2^+^ cells in the VZ‐like zone of the CRTL and ASD organoids at D30. (IMR90‐4: n = 20 VZ‐like regions, NC3‐1: n = 23 VZ‐like regions, NC1B‐3: n = 24 VZ‐like regions, ASD3‐28: n = 23 VZ‐like regions, ASD2‐3: n = 32 VZ‐like regions, ASD1‐4: n = 18 VZ‐like regions, ASD versus CTRL: ***p < 0.001). VZ‐like regions from 3 independent biological replicate experiments were analyzed for each cell line. C) Schematic of how cleavage angles were determined from immunostaining data (Left). Representative immunofluorescence images (Right) of p‐VIM and PHH3 illustrating individual angles of proliferating progenitor cells in the control and ASD D30 organoids. Scale bar: 25 µm. The degree of an individual angle was defined by the angle between the division surface and apical surface. D) Bar and polar area charts showing the distribution and proportion of individual angle degrees in the CRTL and ASD D30 organoids (IMR90‐4: 0–30° was 20.90%, 30–60° was 19.40%, 60–90° was 59.70%; NC3‐1: 0–30° was 23.73%, 30–60° was 25.42%, 60–90° was 50.85%; NC1B‐3: 0–30° was 30.43%, 30–60° was 15.22%, 60–90° was 54.35%; ASD3‐28: 0–30° was 38.78%, 30–60° was 28.57%, 60–90° was 32.65%; ASD2‐3: 0–30° was 40.00%, 30–60° was 18.75%, 60–90° was 41.25%; ASD1‐4: 0–30° was 42.35%, 30–60° was 20.00%, 60–90° was 37.65%; CTRL versus ASD: 0–30°:n = 3 cell lines, **p < 0.01, 30–60°: n = 3 cell lines, ns *p* = 0.60, 60–90°: n = 3 cell lines, **p < 0.01). E) Histograms displaying the distribution and proportion of individual angle degrees in the CRTL and ASD D30 organoids (CTRL versus ASD: 0–30°:n = 3 cell lines, **p < 0.01, 30–60°: n = 3 cell lines, ns *p* = 0.60, 60–90°: n = 3 cell lines, **p < 0.01). (F) UMAP plots of cortical neuron (CN) cluster coloured on 6 cell lines. G) The cortical neurons (CN) were reclustered at D30, and UMAP plots were generated based on cell types. CR: Cajal‐Retzius cells, DL: deep layer cells, IP: intermediate progenitor. H) Feature plots showing differential expression of the selected DL marker *BCL11B*(*CTIP2*), *FEZF2*, *TBR1* and CR marker *RELN* on UMAP plots. I) Dot plots showing the expression of deep layer and CR cell‐related genes (*BCL11B, FEZF2, TBR1* and *RELN*) in “DL1”, “DL2” and “CR cells” clusters in CTRL and ASD groups.

We next performed a 5‐ethynyl‐2′‐deoxyuridine (EDU) assay to confirm the reduction in RG cells and to further elucidate the premature neural differentiation of D30 ASD organoids. The ratio of EDU^+^/SOX2^+^ cells in the ASD organoids was significantly lower than that in the CTRL organoids (**Figure** **3**A,B). Additionally, the percentage of SOX2^+^ cells in the VZ‐like zone was reduced and the diameter of PKCλ^+^ lumens was increased in the ASD organoids compared to the CTRL organoids (Figure S3A,B, Supporting Information). Moreover, the percentages of CTIP2^+^ (a layer V/VI marker) and FOXP2^+^ (a layer VI marker) cells were increased in the ASD organoids compared to the CTRL organoids (Figure S3C, Supporting Information). Gene set enrichment analysis (GSEA) revealed that the regulation of nuclear division and the cell cycle checkpoint term, which represent high cell proliferation activity, were highly enriched in the CTRL group, whereas the regulation of actin filaments and negative regulation of MAP kinase activity terms, which imply high cell differentiation activity, were enriched in the ASD group (Figure S3D and Table S8, Supporting Information). Thus, we used p‐VIM and PHH3, which are located at the apical surface and label mitotic cells, to determine the angle of division that affects RG differentiation (Figure 3C). Compared with those in the CTRL organoids, more RG cells underwent horizontal cleavage with an angle of less than 30° in the ASD organoids, indicating that more ASD RG cells underwent neural differentiation rather than stem cell proliferation (Figure 3D,E).

We next reclustered the cortical neurons (CNs) from the D30 organoid transcriptome data based on the expression of marker genes using UMAP and identified 7 cell types, including CN1/2, Cajal–Retzius (CR) cells, deep layer (DL) 1/2 cells and intermediate progenitor (IP) 1/2 cells (Figure 3F,G). We subsequently calculated the proportion of each cell type. Most cell types, DL1/2 in particular, comprised the largest percentage, ranging from 54.1% to 84.5%, whereas the proportion of IP1/2 cells varied from 8.2% to 17.6%, indicating significant heterogeneity across individuals (Figure S3E and Table S9, Supporting Information). Moreover, a feature plot revealed that the relative expression levels of *BCL11B, FEZF2, TBR1* and *RELN*, which, respectively, label deep layer neurons (*BCL11B, FEZF2*, and *TBR1*) and CR cells (*RELN*), were increased in early‐generated neurons in the ASD group compared with those in the CTRL group (Figure 3H,I and Table S10, Supporting Information). Furthermore, we observed a smaller increase in the expression of *CUX1, POU3F2*, and *SATB2*, which are associated with upper layer cells, in the ASD group (Figure S3F,G, Supporting Information). Taken together, our results indicate that RG cells undergo premature neural differentiation into deep layer neurons compared with control organoids.

### 2.5 FABP7 Knockdown Results in Premature Cortical Differentiation and Mediates Autistic‐Like Behaviors

**Figure 4:**
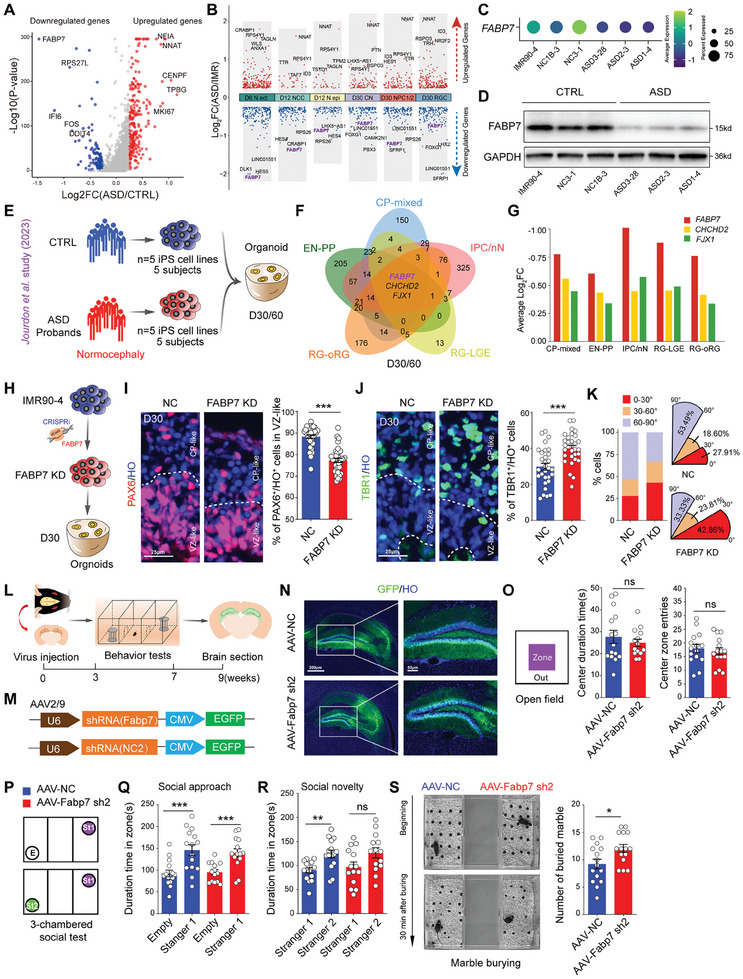
Downregulation of FABP7 results in premature neural differentiation in ASD organoids and mediates autistic‐like behaviors in mice. A) Volcano plots showing differentially expressed genes identified from RGCs in CRTL and ASD organoids at D30. Each red or blue dot denotes an individual gene with *p* ≤ 0.05 and log_2_‐fold change>0.25. B) Differential gene expression analysis showing genes with up‐ and down‐regulated expression across six major cell types from D6 to D30 (D6 N.ect, D12 NCC, D12 N.epi, D30 CN, D30 NPC1/2, D30 RGC). Genes derived from the X or Y sex chromosomes were excluded for further analysis. ChP: choroid plexus, ChP epi:choroid plexus neuroepithelium, CN: cortical neurons, IN: interneurons, NCC: neural crest cells, N. ect: neuroectodermal cells, N. epi: neuroepithelial cells, NPC: neural progenitor cells, oRGC: outer radial glia cells, RGC: radial glia cells, SC: stem cells, VP: ventral progenitors. Each dot denotes an individual gene with *p* ≤ 0.05 and log_2_‐fold change>0.25. C) Dot plots showing the expression of *FABP7* in the CRTL and ASD cell lines at D30. D) Representative western blots of FABP7 expression for the protein level of FABP7 in the CRTL and ASD organoids at D30. E) Schematic diagram showing the generation of cerebral organoids derived from 5 ASD individuals with normocephaly and their unaffected fathers in *Jourdon* et al. study. F) Venn diagram illustrating the intersection of six datasets (CP‐mixed, EN‐PP, IPC/nN, RG‐oRG, and RG‐LGE) sourced from *Jourdon* et al. study, representing the 30/60 days condition. The diagram emphasizes the shared elements among different dataset combinations, with particular attention to the central intersection encompassing the three key genes (FABP7, CHCHD2, FJX1). Radial glia‐ outer radial glia: RG‐oRG, lateral ganglionic eminence radial glia: RG‐LGE, intermediate progenitor cells or newborn neurons: IPC/nN, cortical plate mixed neurons: CP‐mixed, excitatory neurons included early‐born neurons of the preplate: EN‐PP. G) Bar chart depicting the fold change (FC) values of three genes (*FABP7, CHCHD2*, and *FJX1*), with *FABP7* displaying the most significant change. H) Schematic diagram displaying the generation of *FABP7* KD cell lines using CRISPRi technology. I) Representative images of immunostaining for PAX6 in the D30 IMR90‐4 and *FABP7* KD (IMR90‐4) organoids (Left). Scale bar: 25 µm. Histograms (Right) showing the quantification of PAX6^+^/HO^+^ cells in VZ‐like of the NC and *FABP7* KD groups (NC: n = 35 organoids, *FABP7* KD: n = 34 organoids, ***p < 0.001). Organoids from 3 independent biological replicate experiments were analyzed for each cell line. J) Representative images of immunostaining for TBR1 in the D30 IMR90‐4 and *FABP7* KD (IMR90‐4) organoids (Left). Scale bar: 25 µm. Histograms (Right) showing the quantification of TBR1^+^/HO^+^ cells in CP‐like of the NC and *FABP7* KD groups (NC: n = 30 organoids, *FABP7* KD: n = 30 organoids, ***p < 0.001). Organoids from 3 independent biological replicate experiments were analyzed for each cell line. K) Bar and polar area charts showing the distribution and proportion of individual angle degrees in the NC and *FABP7* KD D30 organoids (NC: 0–30° was 27.91%, 30–60° was 18.60%, 60–90° was 53.49%; *FABP7* KD: 0–30° was 42.86%, 30–60° was 23.81%, 60–90° was 33.33%). L) Schedule for behavior tests after virus injection. M) Schematic of adeno‐associated virus (AAV2/9) vectors for expressing *Fabp7* shRNA. N) Immunostaining images displayed GFP expressed in the hippocampus, including the dentate gyrus (DG) and CA, after virus injection. Scale bar: 200 µm (Left), 50 µm (Right). O) Schematic of the open field test (Left) and histograms (Right) presenting the center zone duration time (AAV‐NC: n = 15 mice, AAV‐Fabp7 sh2: n = 15 mice, ns *p* = 0.44) and center zone entries (AAV‐NC: n = 15 mice, AAV‐Fabp7 sh2: n = 15 mice, ns *p* = 0.58) of the mice in the AAV‐NC and AAV‐*Fabp7* sh2 groups. P) Schematic of the 3‐chambered social test. Q) Histograms displaying the duration time of mice approaching the stranger 1 (St1) zone compared with the empty zone (E) in the AAV‐NC (E: n = 15 mice, St1: n = 15 mice, ***p < 0.001) and AAV‐*Fabp7* sh2 (E: n = 14 mice, St1: n = 14 mice, ***p < 0.001) groups. R) Histograms presenting the duration time of mice approaching the stranger 2 (St2) zone compared to the St1 zone in the AAV‐NC (St1: n = 15 mice, St2: n = 15 mice, **p < 0.01) and AAV‐*Fabp7* sh2 (AAV‐NC: n = 14 mice, AAV‐*Fabp7* sh2: n = 14 mice, ns *p* = 0.06) groups. S) Representative optical imaging of marble‐burying tests at the beginning and 30 min later (Left). Histograms (Right) showing the number of marbles buried after 30 min in the AAV‐NC and AAV‐*Fabp7* sh2 groups (AAV‐NC: n = 15 mice, AAV‐ *Fabp7* sh2: n = 15 mice, *p < 0.05).

We first performed an analysis of differentially expressed genes in RG cells between the 3 ASD and 3 CTRL groups at D30 to obtain insights into the molecular mechanism underlying the premature neural differentiation in ASD organoids. We identified a total of 124 genes whose expression was decreased in the ASD group and 232 genes whose expression was increased in the ASD group. The volcano plot displays the five most significantly upregulated and downregulated genes (**Figure** **4**A; Table S10, Supporting Information). Furthermore, we identified the DEGs in the six cell types in the organoids from the IMR90‐4 and ASD3‐28 groups at three developmental time points from D6 to D30 (Figure 4B; Table S7, Supporting Information). We identified ten overlapping genes with downregulated expression and four overlapping genes with upregulated expression in the ASD group (Figure S4A, Supporting Information). By combining time‐series scRNA‐seq and multiple‐cell‐line scRNA‐seq data, we found that the expression of FABP7, which is an essential gene involved in regulating the establishment of RG fibers during early brain development,^[^ ^23^ ^]^ was the top downregulated gene in our study (Figure S4B, Supporting Information) and its expression was significantly decreased in the ASD groups (Figure 4C). Similarly, we measured the levels of the FABP7 mRNA and protein using qRCR and western blotting (WB) assays and discovered a significant reduction in FABP7 expression in the ASD organoids compared with that in the CTRL organoids (Figure 4D; Figure S4C,D, Supporting Information). We performed a WB assay of 4 control (4 subjects) and 6 ASD (6 subjects) cell lines to detect the expression of FABP7 and further confirm our findings and observed that the FABP7 protein levels were both lower in the ASD groups than in the controls (ASD iPSC lines: ASD1‐4, ASD2‐3, ASD3‐28, TASD2A‐1, TASD2B‐1 and TASD3A‐1; control iPSC lines: IMR90‐4, NC3‐1, NC1B‐3 and ihtc‐03) (Table 1 and Figure S4E, Supporting Information).

Recently, *Jourdon* et al. revealed an imbalance of excitatory cortical neuron subtypes in forebrain organoids derived from individuals with macrocephalic and normocephalic autism.^[^ ^1^ ^]^ In their study, they generated iPS cell lines from 5 normocephalic and 8 macrocephalic ASD probands and their corresponding unaffected fathers (Figure 4E). The authors subsequently performed sc‐RNA sequencing of ASD forebrain organoids at both D30 and D60. Here, we downloaded the differentially expressed genes (DEGs) identified at D30 and D60 from the study by *Jourdon* et al. for further analysis. We analyzed the downregulated DEGs of five cell types (radial glia‐outer radial glia: RG‐oRG; lateral ganglionic eminence radial glia: RG‐LGE; intermediate progenitor cells or newborn neurons: IPC/nN; cortical plate mixed neurons: CP‐mixed; and excitatory neurons, including early‐born neurons of the preplate: EN‐PP) at D30 and D60 in normocephalic ASD organoids and identified three overlapping genes (*FABP7, CHCHD2* and *FJX1*) in the ASD groups (Figure 4F), among which the *FABP7* was the most downregulated gene (Figure 4G). Only one overlapping downregulated gene (*FABP7*) was identified among the four cell types (RG‐oRG, IPC/nN, CP‐mixed and EN‐PP) at D30 in normocephalic ASD organoids (Figure S4F, Supporting Information). Importantly, we calculated the relative expression of *FABP7* in both normocephalic and macrocephalic ASD organoids at D30 and D60 and observed that *FABP7* was downregulated in normocephalic ASD organoids but upregulated in macrocephalic ASD organoids (Figure S4G, Supporting Information). These findings strongly indicate that FABP7 might be a promising candidate for ASD diagnosis and treatment.

We clarified whether downregulated *FABP7* expression contributes to the abnormal cortical differentiation of ASD by generating *FABP7* knockdown (KD) IMR90‐4 cell lines using CRISPRi technology (Figure 4H; Figure S4H, Supporting Information). The *FABP7* KD cell line and its corresponding negative control (NC) line were GFP‐positive iPSCs (Figure S4I, Supporting Information), and WB revealed that the FABP7 protein level was dramatically decreased in the *FABP7* KD cell line compared to the control cell line (Figure S4J, Supporting Information). We subsequently conducted immunofluorescence staining for PAX6 and TBR1 in NC and *FABP7* KD organoids at D30. The results revealed that FABP7 deficiency led to a significant decrease in the number of PAX6^+^ cells in the ventricular zone (VZ)‐like zone in the *FABP7* KD groups compared with the NC groups (Figure 4I), whereas the number of TBR1^+^ cells located in the cortical plate (CP)‐like zone was markedly increased in the *FABP7* KD groups compared with the NC groups (Figure 4J). Furthermore, we also detected the proliferation capacity of RGs at D30 using KI67 staining and found that the number of KI67^+^ cells was markedly lower in the VZ‐like zone of *FABP7* KD organoids than in that of CTRL organoids (Figure S4K, Supporting Information). Using p‐VIM and PHH3 staining, we measured the cleavage angle of RGs and found that the percentage of RG cells that underwent horizontal cleavage with an angle less than 30° in the *FABP7* KD organoids was greater than that in the CRTL organoids (Figure 4K; Figure S4L, Supporting Information), similar to the results obtained from the ASD organoids. These findings indicate that the inhibition of FABP7 expression could result in a premature signature similar to the pathological phenotype of ASD organoids.

Previous study have demonstrated that *Fabp7* KO mice displayed a hyperactive and anxiety‐related phenotypes associated with a proportion of schizophrenia and ASD sufferers,^[^ ^30^ ^]^ nevertheless, whether *Fabp7* deficiency results in autistic‐like behaviors, including repetitive stereotyped behaviors and social defects, are still uncertain. Due to in utero electroporation of mouse embryos are hard to do the behavioral tests because of the high lethality and low transfection efficiency,^[^ ^31^ ^]^ nor to ensure the precise electroporated position of plasmids, we tried to knockdown the expression of *Fabp7* in adult mouse hippocampus, where keeps continuous neurogenesis during adult stage, to investigate whether the *Fabp7* KD mice exhibit the autistic‐like behaviors, since previous studies have reported that regulation of several genes in adult hippocampus could cause or rescue autism‐like social defects in mice.^[^ ^32^, ^33^ ^]^ Thus, we synthesized a *Fabp7* KD construct encoded by AAV2/9, which infects both progenitor cells and neurons,^[^ ^34^ ^]^ injected it into the mouse hippocampus and evaluated autistic‐like behaviors 3 weeks after virus infection using the behavioral tests described in previous studies^[^ ^32^ ^]^ to further investigate whether downregulation of *Fabp7* could cause autistic‐like behaviors in mice (Figure 4L). First, we generated U6‐*Fabp7* shRNA encoded by AAV2/9 and measured the level of the Fabp7 mRNA using qPCR, and its expression was notably downregulated in cultured primary mouse cortical progenitors after virus infection (Figure 4M; Figure S5A, Supporting Information). The behaviors of the mice with bilateral virus infection were tested beginning at 3 weeks postinjection. Immunofluorescence staining for GFP revealed that the AAVs specifically infected the hippocampus, including the DG and CA3 regions (Figure 4N). The open field test was performed to measure the movement, exploration and anxious behaviors of the mice. We observed that the *Fabp7* KD mice presented no significant changes in exploration or anxious behaviors, including total movement, duration, number of entries into the center zone, or mean velocity or distance traveled in the zone (Figure 4O; Figure S5B, Supporting Information). Moreover, the novel object recognition and Y maze tests, which detect the curiosity and short‐term memory of the mice, revealed no significant differences between the *Fabp7* KD and NC mice (Figure S5C,D, Supporting Information). For the detection of social behaviors, we used a 3‐chambered social test to assess the social ability of the *Fabp7* KD mice (Figure 4P). We found that both the *Fabp7* KD and control mice spent more time interacting with the Stranger 1 mouse than the empty cage in the social approach test, indicating no marked changes in social approach between the *Fabp7* KD and control mice (Figure 4Q). Interestingly, when we put the Stranger 2 mouse into the empty cage, the subsequent tests revealed that, compared with the control mice, the *Fabp7* KD mice presented a defect in social novelty behavior (Figure 4R). Notably, we determined whether the *Fabp7* KD mice exhibited stereotyped, repetitive behaviors by performing a marble‐burying test and calculated the number of marbles buried by the mice within 30 min (Figure 4S). The results revealed that the *Fabp7* KD mice buried more marbles than the control mice did, which strongly suggested that the *Fabp7* KD mice exhibited autism‐related behaviors.

In summary, we provide evidence that *FABP7* deficiency leads to premature neural differentiation in *FABP7* KD organoids and mediates autistic‐like behaviors in mice.

### 2.6 FABP7 Regulates Premature Differentiation Through a Mechanism Associated with the MAPK Signaling Pathway

**Figure 5:**
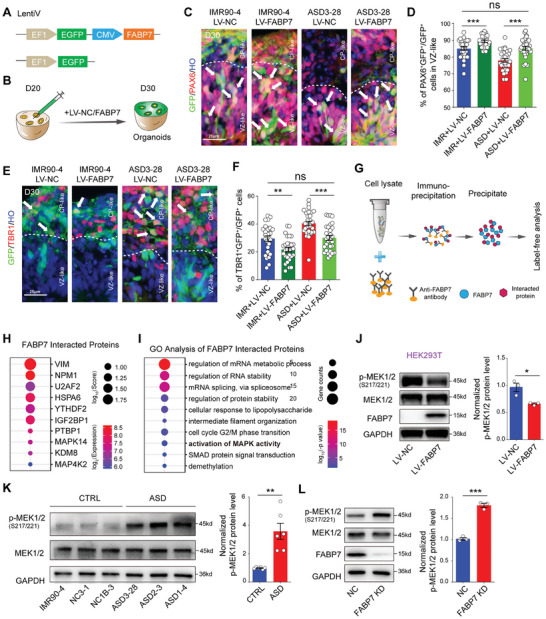
FABP7 regulates premature neural differentiation associated with MAPK signals in ASD organoids. A) Schematic of *FABP7‐EGFP* encoded by lentivirus. B) Schematic of injecting lentivirus encoding *FABP7* or vehicle alone into D20 organoids and then culturing for another 10 days for infection. C) Representative images of immunostaining for PAX6 and GFP in the D30 IMR90‐4 and ASD3‐28 organoids infected with *FABP7* overexpression and control virus, respectively. Scale bar: 25 µm. D) Histograms showing the quantification of PAX6^ + ^GFP^ + ^/GFP^+^ cells in the IMR90‐4 + LV‐NC, IMR90‐4 + LV‐*FABP7*, ASD3‐28 + LV‐NC and ASD3‐28 + LV‐*FABP7* groups. IMR90‐4 + LV‐NC: n = 30 organoids, IMR90‐4 + LV‐*FABP7*: n = 28 organoids, ASD3‐28 + LV‐NC: n = 30 organoids, ASD3‐28 + LV‐*FABP7*: n = 30 organoids, IMR90‐4 + LV‐NC versus IMR90‐4 + LV‐*FABP7*: ***p < 0.001, ASD3‐28 + LV‐NC versus ASD3‐28 + LV‐*FABP7*: ***p < 0.001, IMR90‐4 + LV‐NC versus ASD3‐28 + LV‐*FABP7*: ns *p* = 0.89. Organoids from 3 independent biological replicate experiments were analyzed for each cell line. E) Representative images of immunostaining for TBR1 and GFP in the D30 IMR90‐4 and ASD3‐28 organoids infected with FABP7 overexpression virus and control virus, respectively. Scale bar: 25 µm. F) Histograms presenting the quantification of TBR1^+^GFP^+^/GFP^+^ cells in the IMR90‐4 + LV‐NC, IMR90‐4 + LV‐*FABP7*, ASD3‐28 + LV‐NC and ASD3‐28 + LV‐*FABP7* groups. IMR90‐4 + LV‐NC: n = 29 organoids, IMR90‐4 + LV‐*FABP7*: n = 29 organoids, ASD3‐28 + LV‐NC: n = 30 organoids, ASD3‐28 + LV‐*FABP7*: n = 30 organoids, IMR90‐4 + LV‐NC versus IMR90‐4 + LV‐*FABP7*: **p < 0.01, ASD3‐28 + LV‐NC versus ASD3‐28 + LV‐*FABP7*: ***p < 0.001, IMR90‐4 + LV‐NC versus ASD3‐28 + LV‐*FABP7*: ns *p* = 0.85. Organoids from 3 independent biological replicate experiments were analyzed for each cell line. G) Schematic of protein immunoprecipitation for proteins directly interacting with FABP7. H) Dot plots displaying the average and percent expression of FABP7 binding proteins (selected) in HEK293T cells. I) GO analysis of FABP7‐interacting proteins. GO terms with FDR of less than 0.05 are shown. J) Representative western blotting detection of p‐MEK1/2, MEK1/2 and FABP7 expression in the HEK293T cells after infection with control and FABP7 overexpression lentivirus (Left). Histograms (Right) showing the quantification of p‐MEK1/2 expression in the HEK293T cells after infection with control and FABP7 overexpression lentivirus (NC: n = 3 independent replicates, FABP7 OE: n = 3 independent replicates, *p < 0.05). K) Representative western blots of p‐MEK1/2 and MEK1/2 expression in the CRTL and ASD organoids at D30 (Left). Histograms (Right) showing the quantification of p‐MEK1/2 expression in the CRTL and ASD organoids at D30 (IMR90‐4: n = 2 independent replicates, NC3‐1: n = 2 independent replicates, NC1B‐3: 2 independent replicates, ASD3‐28: n = 2 independent replicates, ASD2‐3: n = 2 independent replicates, ASD1‐4: n = 2 independent replicates, ASD versus CRTL: ***p < 0.01). Organoids (n≥15) from 2 independent biological replicate experiments were analyzed for each cell line. (L) Representative western blotting detection of p‐MEK1/2, MEK1/2 and FABP7 expression in the NC and FABP7 KD organoids at D30 (Left). Histograms (Right) showing the quantification of p‐MEK1/2 expression in the NC and FABP7 KD organoids at D30 (NC: n = 3 independent replicates, FABP7 KD: n = 3 independent replicates, ***p < 0.001).

We constructed a cytomegalovirus (CMV)‐FABP7 plasmid encoded by a lentivirus to further confirm the function of FABP7 in regulating neurogenesis during ASD progression (**Figure** **5**A). We first detected the overexpression of *FABP7* in HEK293T cells (Figure S6A, Supporting Information). Then, we injected the *FABP7*‐overexpressing (OE) lentivirus into CTRL and ASD organoids at D20 and analyzed them at D30 (Figure 5B). Notably, the percentage of PAX6^+^ cells in the *FABP7* OE group was significantly greater than that in the control group of ASD3‐28 cells (Figure 5C,D). Moreover, GFP and TBR1 double staining revealed that the number of GFP^+^TBR1^+^ cells in the *FABP7* OE group was noticeably decreased compared with that in the control group of ASD3‐28 cells (Figure 5E,F).

We conducted a protein coimmunoprecipitation assay to identify FABP7‐interacting proteins and explore the downstream targets of FABP7 involved in regulating cortical neurogenesis. After immunoprecipitation, the separated precipitates of the FABP7‐interacting proteins were subjected to mass spectrometry analysis (Figure 5G; Figure S6B, Supporting Information). A total of 108 FABP7‐interacting proteins were identified as differentially expressed proteins between the FABP7 OE groups and the IgG groups (Table S11, Supporting Information). Notably, we found that MAPK signals (MAPK14 and MAP4K2), which participate in cell self‐renewal and differentiation, were FABP7 target proteins (Figure 5H). The GO analysis revealed that FABP7‐binding proteins are related to the activation of MAPK (Figure 5I). Because MAPK14 was detected in both the FABP7 OE and IgG groups and MAP4K2 is a specific FABP7‐binding protein (Table S12, Supporting Information), we selected MAP4K2 for further study.

MAP4K2 (also known as GCK) is a member of the MAP kinase superfamily and can promote the phosphorylation of components downstream of MAPK, such as MAP2K1 and MAP2K2 (MEK1 and MEK2), to activate their activity through cascade reactions.^[^ ^35^, ^36^ ^]^ We confirmed the MAP4K2 protein level in the ASD organoids by performing a WB assay and found that the level of the MAP4K2 protein did not differ between the ASD and CTRL organoids (Figure S6C, Supporting Information). Next, we tested the levels of phosphorylated and nonphosphorylated MEK1/2 in both HEK293T and SH‐SY5Y cells and found that the levels of phosphorylated MEK1/2 were markedly lower in the FABP7 OE groups than in the control group, whereas the levels of the nonphosphorylated proteins were not affected (Figure 5J; Figure S6D, Supporting Information). We subsequently confirmed the increased level of MEK1/2 phosphorylation in the ASD organoids compared with the CTRL organoids using WB assay and enzyme linked immunosorbent (ELISA) (Figure 5K; Figure S6E, Supporting Information), which could be rescued by overexpressing *FABP7* in the ASD organoids (Figure S6F, Supporting Information). Importantly, we also detected the protein levels of MEK1/2 and p‐MEK1/2 in *FABP7* KD organoids and discovered that the protein level of p‐MEK1/2 was significantly upregulated in *FABP7* KD organoids compared to the NC organoids, whereas the levels of the MEK1/2 proteins were not affected (Figure 5L). Overall, our results suggest that MEK1/2 might mediate the role of FABP7 in regulating premature differentiation during ASD pathogenesis.

### 2.7 MEK2 Overexpression Causes Autistic‐Like Behaviors, and Its Inhibition Restores Premature Neural Differentiation

**Figure 6:**
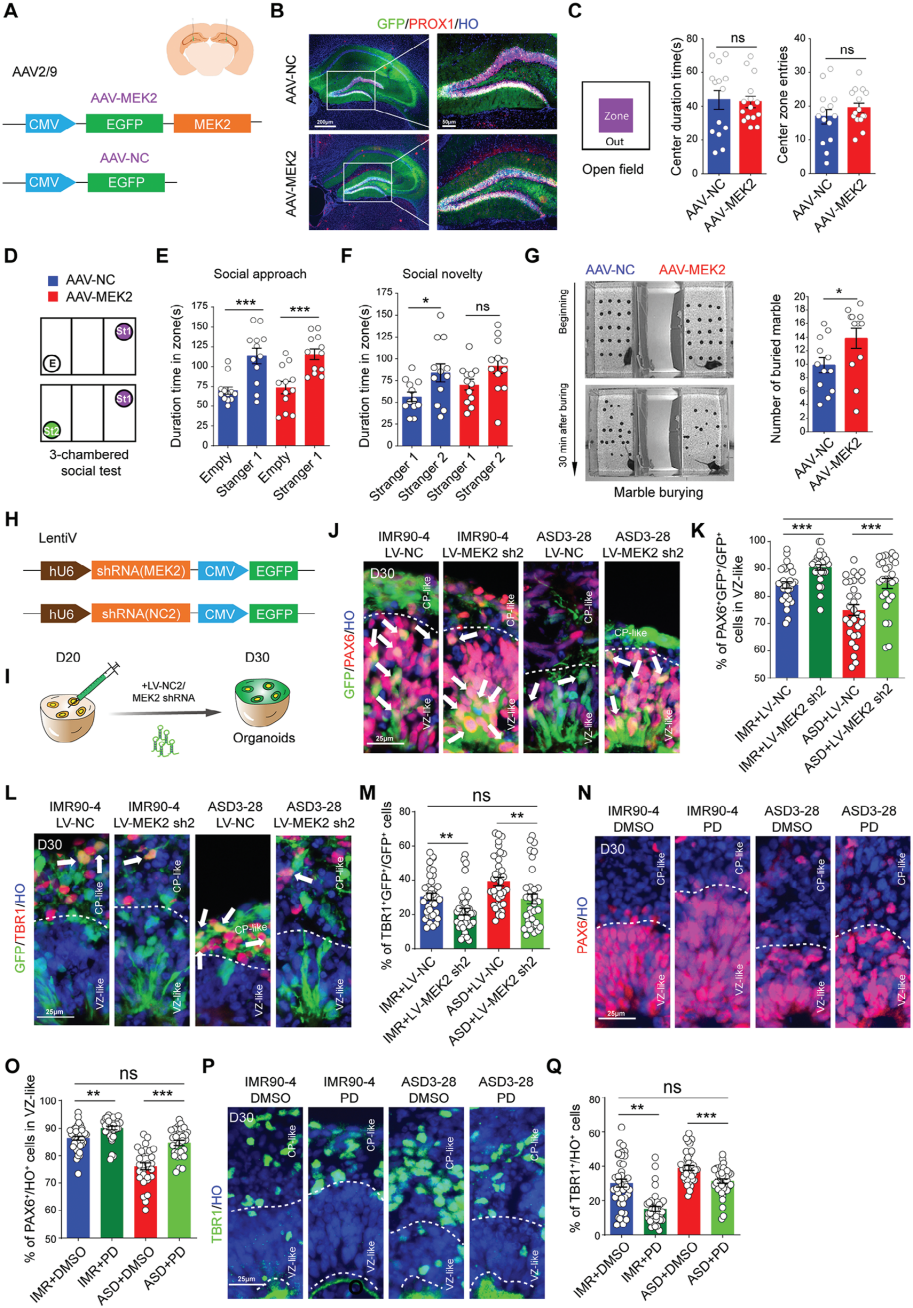
MEK2 overexpression causes autistic‐like behaviors in mice, and its inhibition can rescue premature differentiation in ASD organoids. A) Schematic of AAV2/9 vectors for expressing *MEK2‐EGFP*. B) Immunostaining images displaying GFP expressed in the hippocampus, including the dentate gyrus (DG) and CA, colabeled with the DG granule cell marker Prox1. Scale bar: 200 µm (left), 50 µm (right). C) Schematic of the open field test (Left) and histograms (Right) presenting the center zoneduration time (AAV‐NC: n = 14 mice, AAV‐*MEK2*: n = 15 mice, ns *p* = 0.86) and center zone entries (AAV‐NC: n = 14 mice, AAV‐*MEK2*: n = 15 mice, ns *p* = 0.31) of the mice in the AAV‐NC and AAV‐*MEK2* groups. D) Schematic of the 3‐chambered social test. E) Histograms displaying the duration time of mice approaching the stranger 1 (St1) zone compared with the empty zone (E) in the AAV‐NC (E: n = 12 mice, St1: n = 12 mice, ***p < 0.001) and AAV‐*MEK2* (red) (E: n = 12 mice, St1: n = 12 mice, ***p < 0.001) groups. F) Histograms presenting the duration time of mice approaching the stranger 2 (St2) zone compared to the St1 zone in the AAV‐NC (St1: n = 12 mice, St2: n = 12 mice, *p < 0.05) and AAV‐*MEK2* (red) (AAV‐NC: n = 12 mice, *MEK2* OE: n = 12 mice, ns *p* = 0.06) groups. G) Representative optical imaging of marble‐burying tests at the beginning and 30 min later (Left). Histograms (Right) showing the number of marbles buried after 30 min in the AAV‐NC (blue) and AAV‐*MEK2* (red) groups (AAV‐NC: n = 12 mice, AAV‐*MEK2*: n = 12 mice, *p < 0.05). H) Schematic of lentivirus vectors for expressing shRNA (*MEK2*)‐*EGFP*. I) Schematic overview of injecting lentivirus carrying shRNA targeting *MEK2* into D20 organoids and infecting them for another 10 days. J) Representative images of immunostaining for PAX6 and GFP in D30 IMR90‐4 and ASD3‐28 organoids after knockdown of *MEK2* using lentivirus, respectively. Scale bar: 25 µm. K) Histograms displaying the quantification of PAX6^+^GFP^+^/GFP^+^ cells in the IMR90‐4 + LV‐NC, IMR90‐4 + LV‐*MEK2* sh2, ASD3‐28 + LV‐NC and ASD3‐28 +  LV‐*MEK2* sh2 groups. IMR90‐4 + LV‐NC: n = 31 organoids, IMR90‐4 + LV‐*MEK2* sh2: n = 28 organoids, ASD3‐28 + LV‐NC: n = 30 organoids, ASD3‐28 + LV‐*MEK2* sh2: n = 27 organoids, IMR90‐4 + LV‐NC versus IMR90‐4 + LV‐*FABP7*: ***p < 0.001, ASD3‐28 + LV‐NC versus ASD3‐28 + LV‐*FABP7*: ***p < 0.001, IMR90‐4 + LV‐NC versus ASD3‐28 + LV‐*FABP7*: ns *p* = 0.77. Organoids from 3 independent biological replicate experiments were analyzed for each cell line. L) Representative images of immunostaining for TBR1 and GFP in the D30 IMR90‐4 and ASD3‐28 after knockdown of MEK2 using lentivirus. Scale bar: 25 µm. M) Histograms presenting the quantification of TBR1^+^GFP^+^/GFP^+^ cells in the IMR90‐4 + LV‐NC, IMR90‐4 + LV‐*MEK2* sh2, ASD3‐28 + LV‐NC and ASD3‐28 +  LV‐*MEK2* sh2 groups. IMR90‐4 + LV‐NC: n = 38 organoids, IMR90‐4 + LV‐*MEK2* sh2: n = 41 organoids, ASD3‐28 + LV‐NC: n = 38 organoids, ASD3‐28 + LV‐*MEK2* sh2: n = 35 organoids, IMR90‐4 + LV‐NC versus IMR90‐4 + LV‐*FABP7*: ***p < 0.01, ASD3‐28 + LV‐NC versus ASD3‐28 + LV‐*FABP7*: ***p <0.01, IMR90‐4 + LV‐NC versus ASD3‐28 + LV‐*FABP7*: ns *p* = 0.69. Organoids from 3 independent biological replicate experiments were analyzed for each cell line. N) Representative immunostaining images of PAX6 in the D30 IMR90‐4 and ASD3‐28 control and PD‐rescued organoids. Scale bar: 25 µm. O) Histograms showing the quantification of PAX6^+^/HO^+^ cells in the VZ‐like zone in the MR90‐4 + DMSO, IMR90‐4 + PD, ASD3‐28 + DMSO and ASD3‐28 + PD groups. IMR90‐4 + DMSO: n = 41 organoids, IMR90‐4 + PD: n = 36 organoids, ASD3‐28 + DMSO: n = 30 organoids, ASD3‐28 + PD: n = 30 organoids, IMR90‐4 + DMSO versus IMR90‐4 + PD: **p < 0.001, ASD3‐28 + DMSO versus ASD3‐28 + PD: ***p < 0.001, IMR90‐4 + DMSO versus ASD3‐28 + PD: ns *p* = 0.19. DMSO: dimethyl sulfoxide. VZ‐like regions from 3 independent biological replicate experiments were analyzed for each cell line. P) Representative immunostaining images of TBR1 in the D30 IMR90‐4 and ASD3‐28 control and PD‐rescued organoids. Scale bar: 25 µm. Q) Histograms presenting the quantification of TBR1^+^/HO^+^ cells in the CP‐like zone in the IMR90‐4+DMSO, IMR90‐4 + PD, ASD3‐28 + DMSO and ASD3‐28 + PD groups. IMR90‐4 + DMSO: n = 40 organoids, IMR90‐4 + PD: n = 40 organoids, ASD3‐28 + DMSO: n = 40 organoids, ASD3‐28 + PD: n = 40 organoids, IMR90‐4 + DMSO versus IMR90‐4 + PD: **p < 0.001, ASD3‐28 + DMSO versus ASD3‐28 + PD: ***p < 0.001, IMR90‐4 + DMSO versus ASD3‐28 + PD: ns *p* = 0.64. Organoids from 3 independent biological replicate experiments were analyzed for each cell line.

Given the lower expression of *MEK1* in both the ASD and CTRL organoids (Figure S7A–C, Supporting Information), we deduced that *MEK2*, rather than *MEK1*, might play a key role in regulating cortical differentiation. We constructed *MEK2* OE plasmids encoded by AAV2/9, injected them into the mouse hippocampus and evaluated autistic‐like behaviors 3 weeks after the virus infection via behavioral tests to further investigate whether the activation of MAPK signals could cause autistic‐like behaviors in mice. CMV‐*MEK2* OE constructs are encoded by AAV2/9, and infected cells labeled with GFP and Prox1, a marker for dentate gyrus (DG) granule cells, were detected in the mouse hippocampal DG (**Figure** **6**A,B). The results of the open field test revealed that, compared with the controls, the *MEK2* OE group presented significant increases in total movement and mean velocity (Figure S7D, Supporting Information). However, the mice showed no significant changes in exploratory or anxious behaviors (Figure 6C; Figure S7E, Supporting Information). Similarly, the performance on the Y maze and novel object recognition tests did not differ significantly between *MEK2* OE and NC mice (Figure S7F,G, Supporting Information). Similarly, *MEK2* OE mice also presented social behavioral defects (Figure 6D–F) and stereotyped repetitive behaviors (Figure 6G), similar to did *Fabp7* KD mice, as determined by the 3‐chambered social and marble‐burying tests, which are associated with autistic behaviors.

We inactivated *MEK2* using U6‐shRNA (*MEK2*) encoded by a lentivirus to further determine the role of MEK2 in cortical differentiation in individuals with ASD (Figure 6H). After determining the knockdown (KD) efficiency of the shRNA (*MEK2*) (Figure S7H, Supporting Information), we infected the CTRL and ASD organoids with the *MEK2* sh2 lentivirus to knockdown *MEK2* expression at D20 for 10 days (Figure 6I). The ratio of GFP^+^PAX6^+^/GFP^+^ cells was obviously increased in the *MEK2* KD groups compared to that in the control group in the ASD3‐28 cell line (Figure 6J,K). Additionally, the ratio of GFP^+^TBR1^+^/GFP^+^ cells was significantly decreased in the MEK2 KD group compared with that in the control group in the ASD3‐28 cell line (Figure 6L,M). Moreover, we treated the IMR90‐4 and ASD3‐28 organoids with PD0325901, a MEK inhibitor (MEKi), at D20 for 10 days. We found that the percentage of PAX6^+^ cells in the VZ‐like zone was increased in the PD0325901‐treated groups compared to that in the control group in the ASD3‐28 cell lines (Figure 6N,O). Moreover, the percentage of TBR1^+^ cells was lower in the PD0325901‐treated samples than in the control samples among the ASD3‐28 cells (Figure 6P,Q).

Taken together, these findings indicate that *MEK2* overexpression in the hippocampus causes autism‐related behaviors, whereas MEK2 inhibits could reverse deficits in cortical differentiation in ASD organoids, indicating that the FABP7/MEK pathway may play an important role in the pathogenesis of ASD.

## 3 Discussion

Autism spectrum disorder is a childhood‐onset neurodevelopmental disorder associated with intellectual deficits; however, the molecular mechanism underlying ASD pathogenesis based on convergent pathophysiology remains unclear. Here, we show that the premature differentiation of NSCs over time is regulated by the FABP7/MEK pathway in organoids derived from individuals with idiopathic non‐regressive autism accompanied by normocephaly.

Due to the distinct phenotypes caused by the heterogeneity of genetic and environmental factors,^[^ ^7^, ^20^, ^21^ ^]^ recruiting a cohort with a common clinical diagnosis that might contribute to the discovery of a shared molecular pathway during ASD progression is difficult. In recent decades, investigators have attempted to classify ASD subgroups based on diverse indicators, including the time of onset and head circumference.^[^ ^5^, ^37^ ^]^ Interestingly, an increased head circumference in preschool‐aged boys with ASD is associated with regression.^[^ ^38^ ^]^ From the Jiangsu Birth Cohort Study (JBC), we identified 11 individuals with nonregressive ASD and normocephaly, which coincided with previous conclusions.^[^ ^38^ ^]^ In individuals with macrocephaly and ASD, numerous studies have revealed the hyperproliferation of NSCs and abnormal synaptogenesis in ASD‐derived neurons or organoids.^[^ ^2^, ^10^, ^39^ ^]^ Several genes associated with ASD, such as *FOXG1* and *NOTCH2NL*, have been shown to be involved in the pathology of ASD with macrocephaly.^[^ ^2^, ^15^ ^]^ However, individuals with idiopathic normocephalic autism have rarely been investigated, and little is known about their pathological phenotypes. In this study, we generated patient‐specific iPSCs from individuals with idiopathic normocephalic nonregressive autism and excluded known genetic variants^[^ ^7^ ^]^ and high‐risk environmental factors.^[^ ^20^, ^21^ ^]^ scRNA‐seq data from multiple cell lines and time points revealed the premature differentiation of NSCs at multiple stages, including EBs, rosettes and organoids, in accordance with histomorphological phenotypes in the ASD organoids. We found that the number of NSCs was decreased, and these cells subsequently generated more deep layer cortical neurons. Consistently, reduced generation of NSCs was reported when neurons derived from patients with nonmacrocephalic ASD were used.^[^ ^12^ ^]^ Interestingly, via a pseudotime analysis, *Paulsen* et al. reported that mutations in ASD risk genes in human‐derived organoids resulted in the accelerated maturation of neuron classes,^[^ ^17^ ^]^ whereas in rodent studies, mutations in ASD risk genes, such as *neurofibromatosis type 1* (*NF1*)^[^ ^40^ ^]^ and *DASCAM*,^[^ ^41^ ^]^ resulted in premature neural differentiation^[^ ^40^ ^]^ or spine maturation accompanied by autistic‐like behaviors in mice,^[^ ^41^ ^]^ which implies that premature neural differentiation might be a signature in ASD models. Nevertheless, the mechanism that regulates premature neural differentiation during ASD pathogenesis has not been reported.

Although the pathological phenotypes of ASD with macrocephaly and the regulatory mechanisms involved have been widely elucidated in iPSCs and animal models, investigations of the molecular mechanisms underlying nonregressive ASD in patients with a normal head circumference are lacking. Combined with the scRNA‐seq data, our results confirmed the downregulation of *FABP7* expression in ASD organoids and that *FABP7* KD in normal cell lines led to a premature signature, as observed in ASD organoids, whereas *FABP7* upregulation could normalize the signature in the ASD organoids, indicating a key role in the cortical differentiation of the ASD organoids. Recently, *Jourdon* et al. revealedan imbalance of excitatory cortical neuron subtypes in both normocephalic and macrocephalic autism forebrain organoids.^[^ ^1^ ^]^ By analyzing scRNA‐seq data at D30 and D60 from the study by *Jourdon* et al., we also observed the downregulation of *FABP7* in normocephalic ASD forebrain organoids, consistent with our data. Moreover, previous studies reported that *Fabp7* could maintain the characteristics of neuroepithelial cells and regulate the establishment of radial glial fibers during early cortical development in rodents.^[^ ^23^, ^24^ ^]^ *Fabp7* deficiency promoted neuronal differentiation,^[^ ^24^ ^]^ which was consistent with our findings from cerebral organoids derived from normocephalic individuals with ASD. In addition, as a fatty acid‐binding protein, FABP7 might participate in premature neural differentiation in individuals with ASD by regulating the metabolism of fatty acids, which could be another promising approach for revealing the molecular mechanism of ASD pathology in future studies.

Notably, we explored the downstream target genes of *FABP7* using a protein immunoprecipitation assay and further discovered that MAPK signals, including MEK1/2, mediate the function of FABP7 in regulating premature neural differentiation in ASD organoids. The MAPK signaling pathway has been reported to be involved in various biological processes, including cell self‐renewal and differentiation. The activation of MAPK signals inhibited the self‐renewal of pluripotent embryonic stem cells^[^ ^42^, ^43^ ^]^ and sustained cell cycle arrest,^[^ ^44^, ^45^ ^]^ which supported the hypothesis that the hyperactivation of MAPK signals was responsible for premature neural differentiation in our study. Inactivation of the MAPK cascade via a MEK inhibitor (PD0325901) could rescue the signature in ASD organoids, which might provide an experimental basis for drug discovery for ASD treatment. Furthermore, we inspected whether FABP7 or its novel downstream target genes could serve as risk genes for ASD and cause autistic‐like behaviors in mice by conducting behavioral tests of mice with *Fabp7* KD or *MEK2* OE in the hippocampal DG and observed autistic‐like behaviors in these mice, as predicted.

In summary, we identified premature neurogenesis mediated by the FABP7/MEK pathway in our ASD organoids, which suggests a potential shared molecular mechanism underlying ASD pathogenesis. Our findings might provide novel insights into the molecular mechanism of idiopathic normocephalic ASD pathology with nonregression and promote the development of therapeutic drugs for ASD treatment by targeting the FABP7/MEK axis.

## 4 Experimental Section

### Study Design

This study was designed to investigate the molecular mechanism underlying normocephalic ASD pathogenesis. The study population was drawn from the Jiangsu Birth Cohort Study (JBC), a prospective and longitudinal cohort study. The methods used for recruitment and assignment in this study have been described in detail previously.^[^ ^25^ ^]^ Briefly, the JBC recruited either women who were in their first trimester of natural conception or were about to receive assisted reproduction at the Women's Hospital of Nanjing Medical University or Suzhou Affiliated Hospital of Nanjing Medical University. From September 2018 to April 2019, a total of 513 toddlers reached 2 years of age and were screened for eligibility for this analysis. Toddlers completed the Modified Checklist for Autism in Toddlers screening test (M‐CHAT screening test). A total of 60 of the 513 toddlers were found to be at high risk for ASD. Thirty‐two of the 60 high‐risk toddlers had already undergone additional tests, including the Autism Diagnoses Interview‐R (ADI‐R), Autism Diagnostic Observation Schedule (ADOS) and Diagnostic and Statistical Manual of Mental Disorders‐fifth edition (DSM‐5), with a thorough assessment involving face‐to‐face interaction with toddlers. It was identified 11 toddlers with non‐regressive ASD, 3 of whom were recruited in this study, and collected peripheral blood to construct iPSC lines. Furthermore, the 3 toddlers were confirmed to have no causative genetic variations in 102 ASD risk genes,^[^ ^7^ ^]^ including well‐known genes, such as *CHD8, PTEN, ANK2* and *DSCAM*, as well as ID genes (https://panelapp.genomicsengland.co.uk/panels/285/) used for clinical genetic testing (Table S13, Supporting Information), and no environmental risk factors,^[^ ^20^, ^21^ ^]^ such as maternal type 1 diabetes, advanced maternal age, maternal gestational hypertension and prepregnancy maternal antidepressant use.

An additional 3 toddlers with ASD presenting with normocephaly (TASD2A‐1, TASD2B‐1 and TASD3A‐1) were recruited at The Affiliated Brain Hospital of Nanjing Medical University and completed the Modified Checklist for Autism in Toddlers screening test (M‐CHAT screening test). They were confirmed to have no variations in mutations of 102 ASD risk genes and no environmental risk factors. The Human Research Ethics Committee of Nanjing Medical University reviewed and approved the entire process. Each participant provided written informed consent before inclusion.

Functional studies were conducted in vitro in organoid models and in vivo in mouse models to elucidate whether FABP7 regulates premature neural differentiation in normocephalic ASD organoids and whether FABP7 knockdown in cerebral organoids is associated with MAPK signals and mediates autistic‐like behaviors in mice. For in vitro experiments, EB diameters, rosette radii, lumen perimeters and division angles were measured using ImageJ software. The fluorescence images were processed with ImageJ software for analysis. A double‐blind protocol was used for the collection and analysis of all data. For the behavioral tests, all the experiments were conducted in a quiet and light environment. The behaviors of the mice were monitored and analyzed in a blinded manner using ANY‐maze software. The monitoring data from the mice performing the behavioral tests were excluded from this studies. The number of samples or experimental replicates for each experiment were presented in the figure legends.

### iPSC Reprogramming and Cell Culture

ASD iPSC lines (ASD 1–4, ASD 2–3, ASD 3–28, TASD2A‐1, TASD2B‐1 and TASD3A‐1) were reprogrammed from 6 individuals with normocephalic ASD without significant changes in head circumference using the CytoTune‐iPS 2.0 Sendai Reprogramming Kit (ThermoFisher Scientific). In brief, Sendai viruses containing four reprogramming factors, *SOX2, OCT4, MYC* and *KLF4*, were transduced into monocytes obtained from peripheral blood and cocultured with murine embryonic fibroblasts (MEFs). iPSC clones emerged after 10 days and were cultured with MEFs for 2 passages. Clones were transferred to feeder‐free conditions, in which cells were cultured with Essential 8 (E8) medium (Life Technologies) on plates coated with vitronectin (Thermo Fisher Scientific). The control lines IMR 90–4 (WiCell, no. 17‐W0063), NC3‐1, NC1B‐3 and ihtc‐03 (established in our laboratory) were used in this study. The study was approved by the ethics community of Nanjing Medical University ([2021] No. 83).

For iPSCs, the culture conditions were feeder free, in which iPSCs were maintained in E8 medium on plates coated with vitronectin for 5 to 7 days at 37 °C with 5% CO2. The medium was exchanged with fresh E8 every day until the clones were ≈80% confluent on the plate. For passaging the cells, iPSCs were treated with EDTA (Lonza) at 37 °C for 1 min and then seeded at 1 × 105 cells per well.

HEK293T and SH‐SY5Y cells were cultured in DMEM (Gibco) containing 10% fetal bovine serum (FBS, Gibco) for 5 days before passage at 37 °C with 5% CO2.

For primary mouse cortical progenitors, the cells were isolated from dorsal cerebral cortices around embryonic day 13.5 (E13.5) and cultured in DMEM/F12 (Gibco, 11 320 033) containing 20 ng ml^−1^ bFGF2 (Gibco, PHG0263), 20 ng ml^−1^ EGF (Gibco, PHG0311L) and 2% B27 supplement minus vitamin A (Gibco, 12 587 010) at 37 °C with 5% CO2.

### iPSC Differentiation and Organoid Generation

For the differentiation of organoids, iPSC clones were detached with dispase (1 ml per well, Life Technologies) at 37 °C for 2 min and then resuspended in a mixture of half E8 and half neural induction medium (NIM) in a flask to generate embryoid bodies (EBs), as previously described. EBs were cultured with dual‐SMAD inhibitors, including DMH1 (Tocris) and SB431542 (Tocris), and NIM consisted of DMEM/F12 (Thermo Fisher Scientific), 1% N2 supplement (Thermo Fisher Scientific) and 1% nonessential amino acids (MEM‐NEAA, Thermo Fisher Scientific) for 7 days. On D7, the EBs were attached to the plate at a density of ≈70–100 bodies per well in NIM with 10% FBS for 10 h, and then the medium was exchanged with fresh NIM. The attached EBs were cultured for another 9 days, and rosette structures were observed during this period. On D16, the attached clones were removed by blowing with a 1 ml pipette to form neurospheres (NS) and then maintained in 3D culture with NIM.

### Tissue Dissociation and Single‐Cell RNA Sequencing (scRNA‐seq)

iPSCs (D0), EBs (D6), rosettes (D12) and organoids (D30, D60 and D100) derived from the IMR90‐4 and ASD3‐28 cell lines were prepared for scRNA‐Seq. For iPSC and rosette dissociation, samples were dissociated into a single‐cell suspension by incubation with 1 mL of Accutase (Life Technologies, Thermo Fisher Scientific) for 5 min at 37 °C and washed once with DMEM/F12 for the termination of digestion. Then, the attached iPSCs were suspended in Dulbecco's phosphate‐buffered saline (DPBS) containing 2% FBS using a P1000 pipette. For EB and organoid dissociation, the tissues were incubated with 1 ml of tryp‐LE (Life Technologies, Thermo Fisher Scientific) in 1.5 ml Eppendorf (EP) tubes for 10 min (D6 EBs), 35 min (D30 organoids) or 50 min (D60 or D100 organoids) at 37 °C with mild agitation every 10 min. Digested tissues were terminated by dissociation, washed twice with DMEM/F12 after transient centrifugation and gently dissociated into a single‐cell suspension with 2% FBS in DPBS via a P200 pipette.

The single‐cell suspensions were resuspended in PBS supplemented with 0.05% BSA. Dissociated single cells used for scRNA‐seq were stained with calcein‐AM (Thermo Fisher Scientific, Waltham, MA, USA) and Draq7 (BD Biosciences, New Jersey, USA) to assess viability. Viability was assessed by Trypan blue cell counting and exceeded 90%. The cells (2000—10 000) were loaded in a 10x Genomics Chromium instrument to generate single‐cell GEMs. scRNA‐seq libraries were prepared according to the manufacturer's instructions. The sequencing libraries were quantified with a high‐sensitivity DNA chip (Agilent) using a Bioanalyzer 2100 and a Qubit high‐sensitivity DNA assay (Thermo Fisher Scientific). The library for each sample was sequenced (Illumina, San Diego, CA) with a 150‐bp paired‐end run.

### Single‐Cell RNA‐Seq Data QC and Alignment

It was applied fastp 0.17.0 with default parameter filtering for the adaptor sequence and removed low‐quality reads to obtain qualified clean data.^[^ ^46^ ^]^ UMI‐tools 1.0.0 was used for the single‐cell transcriptomic analysis to identified the cell barcode whitelist.^[^ ^47^ ^]^ The UMI‐based clean data were mapped to the human genome (GRCh38) using STAR 2.7 mapping with customized parameters from the UMI‐tools standard pipeline to obtain UMI counts for each sample.^[^ ^48^ ^]^ Cells containing more than 200 expressed genes with a mitochondrial DNA percentage of less than 30% passed the cell quality filter, which was followed by the deletion of mitochondrial genes from the expression table.

### Single‐Cell RNA‐Seq Data Analysis

The Seurat package (version: 3.2.2, https://satijalab.org/seurat/) was utilized for cell normalization and regression based on the expression table according to the UMI counts of each sample and percentage of mitochondria to achieve scaled data. PCA was constructed based on the large‐scale data, and the top 30 principal components were applied for UMAP construction. Unbiased spatial mapping of all clusters was performed using VoxHunt. The transcriptomic profiles were compared using the BrainSpan transcriptome database. Using the graph‐based cluster method (resolution = 1.6), the unsupervised cell cluster results for the top 30 PCs and subsequently calculated marker genes with the FindAllMarkers function and the Wilcoxon rank‐sum test using the following criteria was acquired: 1. log_2_FC > 0.25; 2. *p* ≤ 0.05; and 3 min.pct>0.1. For the identification of possible cell subtypes, clusters of the same cell type were selected for a repeated cluster analysis and graph‐based clustering.

### Differentially Expressed Genes Identify and Enrichment Analysis

For the identification of differentially expressed genes among samples, FindMarkers with the Wilcoxon rank‐sum test were used with the following criteria: 1. log_2_FC > 0.25; 2. *p* ≤ 0.05; and 3. min.pct > 0.1. To further elucidate the functional implications of these DEGs, Gene Ontology (GO) and Kyoto Encyclopedia of Genes and Genomes (KEGG) enrichment analyses were conducted using the clusterProfiler package (version 3.18.1) with default parameters.

### Developmental Pseudotime Analysis

A trajectory analysis and pseudotemporal ordering of cells with the monocle2 package of R (version 3.4.0) to characterize the potential process of functional changes in cells was performed and determine possible lineage differentiation between all‐time points. It was used differentialGeneTest to identify marker genes by assessing the expression level of each gene across the cells. The most significantly differentially expressed genes were selected from the marker genes at an FDR‐corrected *p* < 0.01. Dimensionality reduction and trajectory construction were performed using the most significantly differentially expressed genes with the default parameters. All the cells were then ordered through pseudotime to infer their differentiation and development.

### CellAlign Analysis

The trajectories of both the control was compared and ASD samples from Day0 to Day 100 using the “interweight” function from the cellAlign package to interpolate the expression data along one trajectory (window size of interpolation: winSz  =  0.1, number of desired interpolated points, numPts  =  200). The resulting matrix was further scaled using the scaleInterpolate function. The interpolated scaled values were used as inputs to align the pseudotimes with the default parameters (sig.calc  =  F, num.perm = 200). The algorithm calculates pairwise distances between ordered points along two trajectories in the gene expression space. cellAlign then finds an optimal path through the matrix of pairwise distances that preserves pseudotime ordering and minimizes the overall distance between the matched cells.

### Gene Enrichment Analysis

KEGG and GO enrichment analyses were performed to characterize the relative activation of a given gene set using the clusterProfiler 3.18.0 package of R (version 3.4.0).

### Specimen Preparation

For EBs and organoids, tissues were treated with 4% paraformaldehyde (PFA) for 2 h and then soaked in 30% sucrose for 2 days. Dehydrated organoids were embedded in OCT compound and sectioned into 7 µm (EB)‐ and 10 µm (organoid)‐thick sections. For adult mouse brains, tissues were treated with 4% PFA for 12 h and soaked in 30% sucrose for 2 days. The dehydrated tissues were subsequently embedded in OCT compound and sectioned into 30 µm thick sections. All the sections were obtained using a Leica cryostat (Leica, CM 1950) and then maintained at −80 °C until use. For the iPSCs and rosettes, the tissues were rinsed with PBS and then fixed with 4% PFA for 30 min. The fixed iPSCs and rosette colonies were immediately used for immunofluorescence staining.

### EdU Assay

This assay was conducted with a BeyoClickTM EdU cell proliferation kit (Beyotime). ASD and CTRL organoids were incubated with EdU (10 µM) at 37 °C for 1 h. Then, the tissues were fixed with 4% PFA for 4 h and soaked in 30% sucrose for 2 days. After being embedded in OCT compound, the organoid tissues were sliced into 10 µm thick sections. The sections were treated with 0.2% Triton X‐100 (Biolink) for 10 min and then incubated with click reaction solution prepared according to the manual provided with the kit for 30 min. Immunofluorescence staining was subsequently performed.

### Immunofluorescence Staining and Image Collection

Tissue sections or fixed colonies were treated with 0.2% Triton X‐100 (Bio‐Link) for 1 h and blocked with 10% donkey serum (Millipore Sigma) for 1 h. The samples were incubated with primary antibodies (**Table** **2**) overnight at 4 °C and then with secondary antibodies (Table 2) for 4 h at 25 °C the next day. After 3 washes, the samples were cover slipped, mounted with Fluoromount‐G mounting solution (Southern Biotech), and used for fluorescence imaging with an Eclipse 80i fluorescence microscope (Nikon) or laser scanning confocal microscope (Zeiss, LSM800). For optical image collection, photos of the iPSCs, EBs and rosette colonies were randomly captured using an inverted microscope (Nikon, TS100).

**Table 2:** Primary and secondary antibodies used in immunofluorensence staining and western blotting assay.

Anyibody	Isotype	Dilution	Source	Cat.NO.
CTIP2	Rat IgG	1:500(IF)	abcam	ab18465
DCX	Rabbit IgG	1:1000(IF)	Cell Signaling	4604
FABP7	Rabbit IgG	1:2000(WB) 1:500(IP)	Cell Signaling Technology	13347S
FOXG1	Rabbit IgG	1:1000(IF)	abcam	ab18259
FOXP2	Rabbit IgG	1:1000(IF)	abcam	ab16046
GAPDH	Mouse IgG	1:5000(WB)	affinity	T0004
GFP	Chicken IgG	1:1000(IF)	Millipore	ab16901
GFP	Rabbit IgG	1:1000(IF)	Chemicon	AB3080
KI67	Rabbit IgG	1:500(IF)	Invitrogen	180191Z
MAP4K2	Rabbit IgG	1:2000(WB)	abcam	ab184169
MEK1/2	Mouse IgG	1:1000(WB)	Cell Signaling Technology	4694S
NANOG	Goat IgG	1:1000(IF)	R&D Systems	AF1997
NESTIN	Goat IgG	1:1000(IF)	Santa Cruz	SC‐21247
PAX6	Rabbit IgG	1:500(IF)	convance	PRB‐278P
PHH3	Rat IgG	1:1000(IF)	Cell Signaling Technology	9706S
PKCλ	Mouse IgG	1:1000(IF)	BD	610 207
p‐MEK1/2 (S217/221)	Rabbit IgG	1:1000(WB)	Cell Signaling Technology	3958S
PROX1	Rabbit IgG	1:300(IF)	abcam	ab101851
p‐Vimentin	Mouse IgG	1:1000(IF)	MBL	D076‐3
SOX2	Goat IgG	1:500(IF)	R&D Systems	AF2018
TBR1	Rabbit IgG	1:500(IF)	abcam	ab31940
γ‐tublin	Mouse IgG	1:1000(IF)	abcam	ab11316
Alexa Fluor 488	Goat anti‐Chicken IgG	1:1000(IF)	Invitrogen	A11039
Alexa Fluor 488	Donkey anti‐Rat IgG	1:1000(IF)	Invitrogen	A21208
Alexa Fluor 488	Donkey anti‐Rabbit IgG	1:1000(IF)	Invitrogen	A21206
Alexa Fluor 488	Donkey anti‐Mouse IgG	1:1000(IF)	Invitrogen	A21202
Alexa Fluor 488	Donkey anti‐Goat IgG	1:1000(IF)	Invitrogen	A11055
Alexa Fluor 546	Donkey anti‐Rabbit IgG	1:1000(IF)	Invitrogen	A10040
Alexa Fluor 546	Donkey anti‐Mouse IgG	1:1000(IF)	Invitrogen	A10036
Alexa Fluor 546	Goat anti‐Rat IgG	1:1000(IF)	Invitrogen	A11081
Alexa Fluor 546	Donkey anti‐Goat IgG	1:1000(IF)	Invitrogen	A11056
Alexa Fluor 647	Donkey anti‐Mouse IgG	1:1000(IF)	Invitrogen	A31571
Alexa Fluor 647	Donkey anti‐Rabbit IgG	1:1000(IF)	Invitrogen	A31573
Alexa Fluor 647	Donkey anti‐Goat IgG	1:1000(IF)	Invitrogen	A21447
Hoechst332258		1:1000(IF)	Invitrogen	A1339
HRP	Goat Anti‐Mouse IgG	1:5000(WB)	Multi Sciences	70‐GAM0072
HRP	Goat Anti‐Rabbit IgG	1:5000(WB)	Multi Sciences	70‐GAR0072

### Western Blotting and Enzyme Linked Immunosorbent Assay

The samples were lysed with a mixture of radioimmunoprecipitation assay (RIPA) lysis buffer and a protease inhibitor cocktail (Roche). For western blotting (WB) assay, SDS‐PAGE solution was added to the lysate, which was boiled for 10 min before being loaded onto precast SDS‐polyacrylamide gels (GenScript). The proteins were separated via electrophoresis at 120 V and transferred to polyvinylidene fluoride membranes for 2 h at 300 mA. The membranes were subsequently blocked with 5% milk for 1 h. Primary antibodies (MEK, P‐MEK, FABP7 and GAPDH; detailed information was listed in Table 2) were incubated with the membranes at 25 °C for 12 h, after which the membranes were washed with TBST 6 times. The membranes were incubated with secondary antibodies (HRP‐conjugated IgG) (Table 2) for 1 h and then washed with Tris‐buffered saline containing Tween (TBST) 3 times. An ECL system (Bio‐Rad, Gel Doc XR) was used for detection. For enzyme linked immunosorbent assay (ELISA), the protein levels of p‐MEK in the cell lysate were analyzed by ELISA using a human p‐MEK ELISA kit (MM‐61111H2, MEIMIAN, China). The OD_450nm_ values were determined by a microplate reader (Infinite M200 Pro NanoQuant, TECAN, Austria).

### Real‐Time qPCR

Total RNA was extracted from the ASD and CTRL organoids with a TRIzol kit (Thermo Fisher Scientific). The isolated RNA was reverse transcribed into cDNA using a PrimeScriptTM RT reagent kit (TaKaRa) and prepared for qPCR using the SuperScript III First‐Strand Synthesis System (Thermo Fisher Scientific).The primers for qPCR used were as follows: *FABP7* forward primer,

5′‐TCTCACCTCCTTCCTTCTTCT‐3′ and reverse primer, 5′‐AGAACCTTGCCAGTGATGTATT‐3′; *MEK2* forward primer, 5′‐CTCACCATCAACCCTACCATC‐3′, and reverse primer, 5′‐GCAGGTCCACCAGGTTT‐3′; *GAPDH* forward primer, 5′‐TCGACAGTCAGCCGCATCTTCTTT‐3′ and reverse primer, 5′‐ACCAAATCCGTTGACTCCGACCTT‐3′.

### Construction of the FABP7 KD iPSC Line

The FABP7 KD iPSC line was generated as previously described. Briefly, the CRISPRi dual vectors CBh‐dCAS9‐KRAB‐MeCP2:T2A:Hygro (VB010000) and hU6‐sgRNA2#‐hPKG‐EGFP:T2A:Puro (VB220816), encoded by lentiviruses, were designed and synthesized by VectorBuilder (Yunzhou Biosciences, Guangzhou). The designed sgRNA targeted the DNA region from –300 to 50 bp around the transcription start site (TSS) of the *FABP7* gene (NM_0 013 19 039.2). The sequences of sgRNA were used as follows: IMR90‐4‐FABP7‐sgRNA3#: 5′‐TATGCCTGAGAAAGCTCGGC‐3′, non‐targeting sgRNA: 5′‐GCACTACCAGAGCTAACTCA‐3′.

The FABP7 KD cell line was generated by first infecting IMR90‐4 cells with dCas9‐KRAB encoded by a lentivirus for 24 h at 3 days after mechanical passage. Hygromycin was subsequently added to the E8 medium at a concentration of 100 µg ml^−1^ and incubated with the cells for 5 days. The IMR90‐4 iPSC colonies were then cultured with 50 µg ml^−1^ hygromycin to maintain selection until stable colonies appeared. Second, lentiviruses expressing either targeting or scrambled sgRNA were used to infect selected IMR90‐4 iPSC colonies for 48 hours before the replacement of E8 medium supplemented with a ROCK inhibitor (STEMCELL Technologies). Finally, FABP7‐KD iPSC colonies were selected based on EGFP expression and identified by immunofluorescence staining and WB before being used for further experiments.

### Viral infection and MEK inhibitor treatment

The viruses pSLenti‐EF1‐EGFP‐P2A‐Puro‐CMV‐FABP7‐3xFLAG‐WPRE (LV‐FABP7 OE) (viral: 7.21 × 10^8^ particles ml^−1^), pSLenti‐EF1‐EGFP‐P2A‐Puro‐CMV‐MCS‐3xFLAG‐WPRE (LV‐NC) (viral: 6.34 × 10^8^ particles ml^−1^), pSLenti‐hU6‐shRNA (MAP2K2)‐CMV‐EGFP‐F2A‐Puro‐WPRE (LV‐MEK sh) (sh1 viral: 3.42 × 10^8^ particles ml^−1^; sh2 viral: 3.61 × 10^8^ particles ml^−1^; sh3 viral: 4.40 × 10^8^ particles ml^−1^), pSLenti‐hU6‐shRNA (NC2)‐CMV‐EGFP‐F2A‐Puro‐WPRE (LV‐NC2) (viral: 8.11 × 10^8^ particles ml^−1^), pAAV2/9‐U6‐shRNA (Fabp7 2#)‐CMV‐EGFP‐WPRE (AAV‐ Fabp7 sh2) (viral: 4.12 × 10^12^ particles ml^−1^) and pAAV2/9‐U6‐shRNA (NC2)‐CMV‐EGFP‐WPRE (AAV‐NC) (viral: 1.75 × 10^13^ particles ml^−1^), pAAV2/9‐CMV‐EGFP‐P2A‐MAP2K2‐3xFLAG‐WPRE (AAV‐MEK2 OE) (viral: 7.89 × 10^12^ particles ml^−1^) and pAAV2/9‐CMV‐EGFP‐P2A‐3xFLAG‐WPRE (AAV‐NC) (viral: 1.99 × 10^13^ particles ml^−1^) were used in this study (Obio Technology, Shanghai). For HEK293T cells, 20 µl of LV‐FABP7 and 20 µl of LV‐NC were added to the culture medium with polybrene (1:2000) in each well of 6‐well plates and cultured for 6 days before collection. For the organoids, 0.5 µl of LV‐FABP7, 0.5 µl of LV‐NC, 0.8 µl of LV‐MEK sh2 and 0.25 µl of LV‐NC2 were separately injected into each organoid, after which the organoids were cultured in NIM supplemented with polybrene (1:2000) for 10 days. For primary mouse cortical progenitors, 20 µl of AAV‐Fabp7 sh2 and 5 µl of AAV‐NC were added to the culture medium with polybrene (1:2000) in each well of 6‐well plates and cultured for 6 days before collection.

For MEK inhibitor treatment, a MEK inhibitor (MEKi, PD0325901, Sigma–Aldrich) was dissolved in DMSO at a concentration of 10 mM. The solution was added to NIM at a final concentration of 5 µM and cocultured with D20 organoids for 10 days.

### Protein Immunoprecipitation (CoIP) and Mass Spectrometry

Six days after LV‐FABP7 infection, the cultured cells were dissociated with 1 ml of EDTA (Lonza) and transferred to 1.5 ml EP tubes. After centrifugation at 1000 rpm, the precipitated cells were treated with cell lysis buffer (a mixture of RIPA buffer and cocktail) in an ice bath for 30 min and lysed using an intelligent ultrasonic processor (ATPIO, Φ2; 3 s on, 3 s off, 10 cycles). The lysate was centrifuged at 13 000 rpm for 15 min, and the supernatant was collected for further use. For immunocomplex formation, a cell lysate containing 400 µg of total protein was incubated with anti‐FABP7 or IgG antibodies overnight at 4 °C, while a cell lysate containing 40 µg of total protein was separated as the input. Agarose beads were added to the immunocomplexes and incubated for 1 h, after which the beads were washed 3 times with lysis buffer and concentrated at 3000 rpm 3 times. The beads were transferred to 40 µl of SDS buffer and boiled for 15 min. After transient centrifugation, the supernatant was loaded onto precast SDS polyacrylamide gels and the proteins were separated via electrophoresis at 120 V.

For mass spectrometry analysis, the potential targeted bands within LV‐FABP7 or IgG polyacrylamide gel lanes were cut into small pieces after silver staining,^[^ ^49^ ^]^ and sent to the Beijing Bio‐Tech Pack Technology for further analysis. Briefly, the gels were washed 3 times with ultrapure water and fixed using ethanol/acetic acid/water mixtures. After 2 washes with 20% ethanol, the gels were immersed in sensitizer solution (0.8 mM sodium thiosulfate) for 1 min. Then, the gels were submerged in a 0.2% silver nitrate solution and shaken gently for 30 min. The basic developer solution, which contained potassium carbonate, formaldehyde and sodium thiosulfate, was subsequently added, and the samples were carefully observed. When the protein bands were sufficiently stained, the staining was terminated by transferring the gels into Tris‐HAc solution.

### Animals and Virus Injection

C57BL/6 mice (male, 8 weeks) were purchased from GemPharmatech (Nanjing, Jiangsu, China) and reared in the Animal Laboratory Resource Facility at Nanjing Medical University. All animal experiments in this study followed protocols and received permission from the Animal Care and Use Committee of Nanjing Medical University (IACUC‐2104023). The mice were housed on a 12‐h light/dark cycle in a room. Virus injection into the mouse hippocampus was performed according to previously described protocols with modifications. The mice were fixed on a stereotaxic instrument and anesthetized with 1.5% isoflurane mixed with oxygen. AAV‐*Fabp7* sh2 (0.5 µl), AAV‐NC virus (0.2 µl), AAV‐*MEK2* OE (0.5 µl) and AAV‐NC virus (0.2 µl) were stereotaxically injected into the hippocampal dentate gyri of mice at certain coordinates (bregma, X, Y, Z = ‐2.3, ±2.1, ‐2.1) at a rate of 0.1 microliters per minute. Before removal, the needle was left in place for 10 min to prevent reflux of the virus. After virus injection, the mice were housed in the animal facility for 3 weeks before the behavioral tests.

### Behavioral Tests

Three weeks after the virus injection, the C57BL/6 mice (male, 11 weeks) were placed in the test room for at least 1 h before each experiment. Seventy percent ethanol was used to clean the apparatuses before the test was started for each mouse.

### Open Field Test

The subject mouse was placed at a corner of the test box (50 cm × 50 cm × 50 cm) and allowed to explore the environment freely for 15 min. The behavior of the tested mouse was recorded by a hanging camera. The time spent in the center zone (25 cm × 25 cm × 25 cm) and outer zone, the number of crossings of the boundary of the center arena, and the total traveled distance were quantitatively analyzed.

### Y‐Maze

The apparatus used in this experiment was a Y‐shaped maze (25 cm × 5 cm× 15 cm) consisting of three arms at angles of 120 degrees. In the memory acquisition stage, the tested subjects were placed in the center of the maze with one randomly closed arm serving as the novel arm and allowed to explore the environment freely for 10 min. After a 1 h interval, the novel arm was opened, and the tested mice were randomly placed in one of the other two arms. The tested subjects were allowed to explore the environment freely for 10 min. When the animals explored a novel arm that was different from the previous arm from which they exited, we considered them to have entered a novel zone. The number of entries and duration spent in the novel zone were recorded.

### Novel Object Recognition Test

This test had 3 phases. In the first phase, the mice were allowed to explore the open‐field apparatus (50 × 50 × 50 cm) in the absence of objects for 5 min. Two identical objects were subsequently placed in the test box, and the mice were allowed 10 min to become familiar with them in the second phase. In the last phase, one of the identical objects was replaced with a novel object (NO), and the time that the subject mouse spent around the new object was recorded.

### Three‐Chambered Social Test

The test apparatus comprised three equal‐sized rectangular boxes (20 × 40 × 22 cm). Age‐matched C57BL/6 male mice served as Stranger 1 (St1) or Stranger 2 (St2) animals. The subject mouse was placed in the middle chamber and allowed to explore the environment for 5 min. In the social approach stage, the Stranger 1 mouse was randomly placed in one empty wire cage in one of the side chambers, and the subject mouse was allowed to explore all three chambers freely for 10 min. In the social novelty stage, the Stranger 2 mouse was placed in another empty wire cage in the other side chamber for 10 min. The time that the experimental mouse spent in each chamber was measured to qualify its sociability.

### Marble‐Burying Test

The experimental apparatus used three equal‐sized rectangular boxes (20 × 40 × 22 cm) covered with 5 cm deep bedding in two side boxes. An opaque partition was used to avoid mutual interference of each subject. Twenty marbles (4 × 5, 15 mm diameter) were placed gently on bedding before the experiment. The subject mouse was placed in the box for 30 min, and the number of buried marbles (at least 1/2 covered) was counted.

### Quantification and Statistical Analysis

All data shown in this article were analyzed by GraphPad Prism 9.0 and were presented as the mean ± SEM. Student's t test was performed for comparison of two groups. *p* values < 0.05 were considered to indicate a significant difference.

### Data Availability

The raw scRNA‐Seq data used in this study have been deposited in the NCBI Sequence Read Archive (SRA) (accession number PRJNA826352) and Gene Expression Omnibus (GEO) at https://www.ncbi.nlm.nih.gov/geo/. The accession number is GSE203201. Publicly available data used in this paper include the BrainSpan transcriptome database^[^ ^50^ ^]^ and human embryonic PFC single‐cell transcriptome.^[^ ^51^ ^]^

## Conflict of Interest

The authors declare no conflict of interest.

## Author Contributions

X.H., Y.H., and Y.W. contributed equally to this work. Z.H. and Y.L. initiated, conceived and supervised the study. X.H., Y.H. and Y.W. prepared the manuscript. L.H. and Y.H. collected and analyzed Jiangsu Birth Cohort (JBC) data with Y.L., H.M., H.S. and X.K. X.H. and Y.W. reprogrammed iPSCs from ASD individuals and generated organoids. X.H., Y.H. performed RNA‐Seq and scRNA‐Seq analyses with Y.H., X.Z. and Y.G. X.H., Y.W. performed immunofluorensence staining, WB, qPCR and EDU assay with WH. Y.W. performed organoids virus injection and further data analyses with CC. X.H., Y.W. and Y.H. performed protein immunocoprecipitation assay. W.H., X.H. and L.H. performed animal behavior tests and further data analyses. All authors critically reviewed and provided feedback on drafts and approved of the final version.

## Supporting information



Supporting Information

Supplemental Table 1‐13

## Data Availability

The data that support the findings of this study are available from the corresponding author upon reasonable request.
