# Supplementary material for: Deficiency of FABP7 Triggers Premature Neural Differentiation in Idiopathic Normocephalic Autism Organoids
Source: Adv Sci (Weinh). 2024 Nov 18;12(2):2406849. doi: 10.1002/advs.202406849 (PMC11727249; doi:10.1002/advs.202406849)
Supplement: Supplementary file 1 — Supporting Information [file ADVS-12-2406849-s002.docx]

**Supporting Information**

**Deficiency of FABP7 triggers premature neural differentiation in** **idiopathic normocephalic autism organoids**

Xiao Han^1,2,^^5,†^ , Yuanlin He^1,3,5,†^, Yuanhao Wang^1,2,†^, Wenzhu Hu^3^, Chu Chu^2^, Lei Huang^1,3^, Yuan Hong^2^, Lu Han^4^, Xu Zhang^1^, Yao Gao^3^, Yuan Lin^1,6^, Hongxia Ma^1,3^, Hongbing Shen^3^, Xiaoyan Ke^4^, Yan Liu^1,2,5,^*, Zhibin Hu^1,3,5,^*

^1^Interdisciplinary InnoCenter for Organoids, State Key Laboratory of Reproductive Medicine and Offspring Health, Nanjing Medical University, Nanjing, Jiangsu, China.

^2^Institute of Stem Cell and Neural Regeneration, School of Pharmacy, Nanjing Medical University, Nanjing, Jiangsu, China.

^3^Department of Epidemiology and Biostatistics, Center for Global Health, School of Public Health, Nanjing Medical University, Nanjing, Jiangsu, China.

^4^ Autism Research Center, State Key Laboratory of Reproductive Medicine, The Affiliated Brain Hospital of Nanjing Medical University, Nanjing, China.

^5^State Key Laboratory of Reproductive Medicine (Suzhou Centre), The Affiliated Suzhou Hospital of Nanjing Medical University, Suzhou Municipal Hospital, Gusu School, Innovation Center of Suzhou, Nanjing Medical University, Suzhou, Jiangsu, China.

^6^Department of Maternal, Child and Adolescent Health, School of Public Health, Nanjing Medical University, Nanjing, China.

† These authors contributed equally: Xiao Han, Yuanlin He and Yuanhao Wang.

* Co-corresponding author: [yanliu@njmu.edu.cn](mailto:yanliu@njmu.edu.cn) and [zhibin_hu@njmu.edu.cn](mailto:zhibin_hu@njmu.edu.cn)


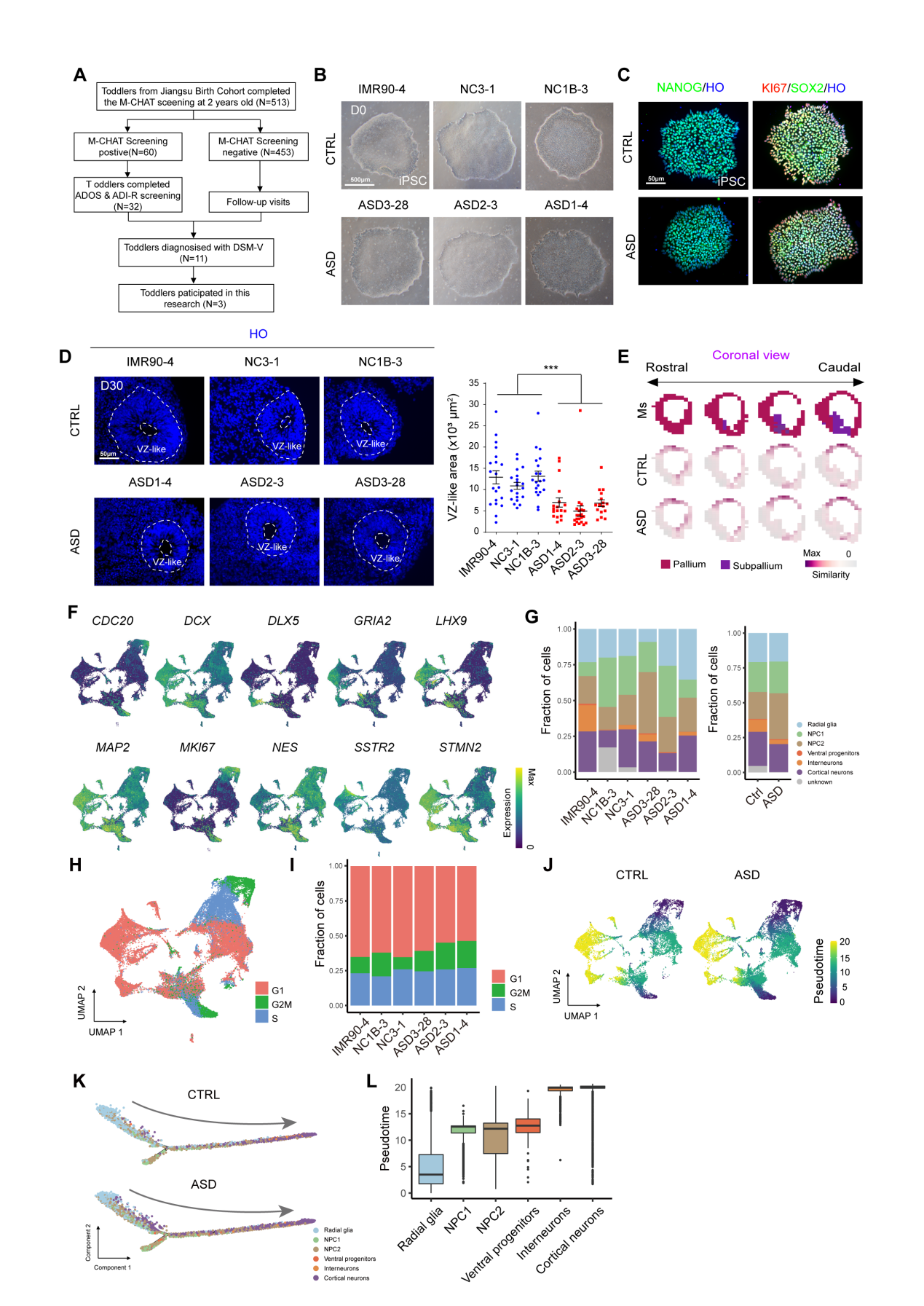


**Figure S1. Identification of pluripotency and scRNA-seq analysis of CRTL and ASD cerebral organoids at D30**

1. Flowchart of ASD screening and diagnosis for toddlers studied in a prospective birth cohort.
2. Optical imaging of CTRL and ASD iPS cell lines. Scale bar: 500μm.
3. Immunofluorensence staining of pluripotency marker NANOG, stem cell marker SOX2 and proliferation marker KI67 for CTRL and ASD iPS cells. Scale bar: 50μm.
4. Representative image of the CTRL (IMR90-4, NC1B-3 and NC3-1) and ASD (ASD3-28, ASD2-3 and ASD1-4) organoids labelled by Hoechst (HO) at D30 (Left). Scale bar: 50 μm. Dot graphs (Right) displaying the area of the VZ-like in the CTRL and ASD organoids (IMR90-4: n=20 organoids, NC1B-3: n=20 organoids, NC3-1: n=20 organoids, ASD3-28: n=16 organoids, ASD2-3: n=21 organoids, ASD1-4: n=18 organoids, ASD versus CTRL: *** p<0.001). Organoids from 3 independent biological replicate experiments were analyzed for each cell line.
5. VoxHunt spatial brain mapping of all the scRNA-seq data of the CRTL and ASD organoids at D30 onto data from the E13.5 mouse brain from the Allen Brain Institute. Coronal views are displayed with scaled similarity scores. Max, maximum.
6. UMAP plots coloured on gene expression of representative genes used to assign cluster identities (related to Figure 1H).
7. The chart showing the comparisons of cell composition between CRTL and ASD cell lines at D30 (Left: each cell lines; Right: CRTL vs ASD).
8. UMAP plots coloured on cell cycle phase (G1, G2/M, S).
9. The chart displaying the comparisons of cell cycle phase between CRTL and ASD cell lines at D30.
10. UMAP plot of all cells in the CRTL and ASD oragnoids at D30 colored by pseudotime. Cells appearing earliest in pseudotime are denoted by dark purple, and those latest in pseudotime are in yellow.
11. Differentiation trajectory generated using Monocle 2 with all cell types excepted unknown cells in ASD and control organoids. Radial glia cells were present at the start point and the cortical neurons were present at the end point of the pseudotime trajectory. The trajectory is similar between ASD and control. Cells were colored by cell types.
12. Boxplot showing the distribution of pseudotime within each cell type at D30 between ASD and CTRL groups.


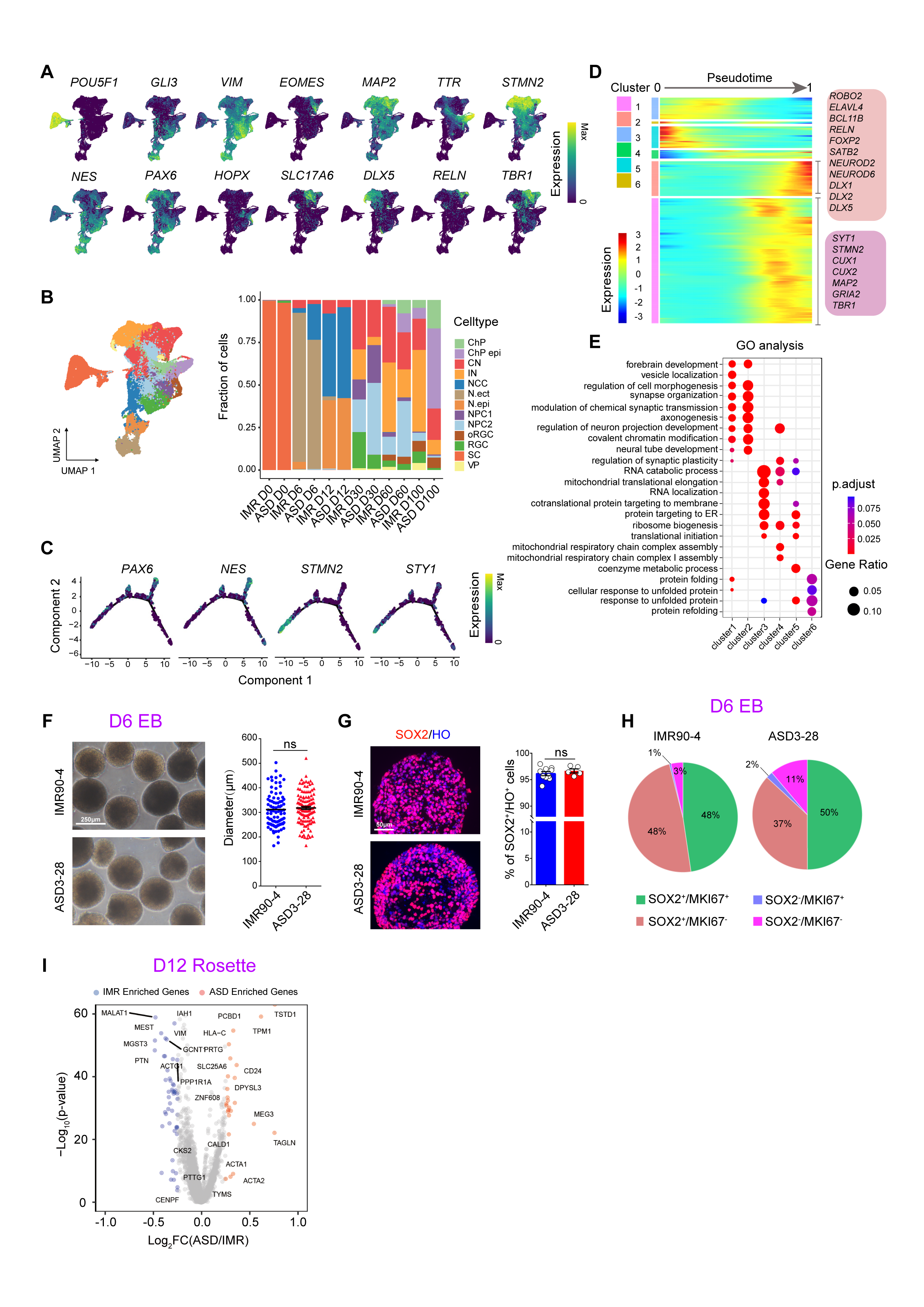


**Figure S2. Time series scRNA-seq analysis of CRTL and ASD cerebral organoids**

1. UMAP plots coloured on gene expression of representative genes used to assign cluster identities.
2. UMAP plot of cell types annotated according to expression of known marker genes in IMR90-4 (n=6) and ASD-3-28 (n=6) over six developmental time pionts. ChP: choroid plexus, ChP epi: choroid plexus neuroepithelium, CN: cortical neurons, IN: interneurons, NCC: neural crest cells, N. ect: neuroectodermal cells, N. epi: neuroepithelial cells, NPC: neural progenitor cells, oRGC: outer radial glia cells, RGC: radial glia cells, SC: stem cells, VP: ventral progenitors. Chart on the right shows the comparisons of cell composition between the IMR90-4 and ASD3-28 from D0 to D100.
3. Expression levels of early neural differentiation markers (*NES*, and *PAX6*) and mature neural markers (*STMN2*, and *STY1*) along the lineage trajectory.
4. Expression of genes with significantly differential trajectories over pseudotime in IMR90-4 and ASD3-28 cells by Monocle2 (qval<10-10). Trajectories grouped by unsupervised hierarchical clustering are centered across genes. Genes in significantly enriched regulation of neuron projection development, axonogenesis, glutamate receptor signaling pathway as well as synapse organization are highlighted in cluster1 and cluster2.
5. GO analysis of six cluster genes from pseudotime trajectory. The color of dots indicates high (red) or low (blue) enrichment. The size of dots displayed the overlap between the input gene lists with the collection of gene sets.
6. Optical imaging of IMR90-4 and ASD3-28 EBs at D6. Scale bar: 250μm. Dot graph displaying the diameter of the IMR90-4 and ASD3-28 EBs (IMR90-4: n=89 EBs, ASD3-28: n=115 EBs, ns: no significance, p=0.5038). EBs from 3 independent biological replicate experiments were analyzed for each cell line.
7. Immunofluorescence staining of SOX2 for the CTRL and ASD EBs at D6 (Left). Scale bar: 50 μm. Histograms (Right) showing that the ratio of SOX2^+^/HO^+^ cells is not changed between the IMR90-4 and ASD3-28 EBs (IMR90-4: 96.57 ± 0.2969, n=23 EBs; ASD3-28: 97.08 ± 0.2379, n=18 EBs. ns: no significance, p=0.2097). EBs from 3 independent biological replicate experiments were analyzed for each cell line.
8. Pie charts showing the ratio of marked cells in the neuroectodermal cluster from D6 scRNA-seq outcomes. Green represents SOX2^+^/MKI67^+^ cells (IMR90-4: 48%, ASD3-28: 50%), red represents SOX2^+^/MKI67^-^ cells (IMR90-4: 48%, ASD3-28: 37%), pink represents SOX2^-^/MKI67^+^ cells (IMR90-4: 3%, ASD3-28: 11%) and light blue represents SOX2^-^/MKI67^-^ cells (IMR90-4: 1%, ASD3-28: 2%).
9. Volcano plots showing differentially expressed genes identified from neuroepithelial cells in ASD3-28 and IMR90-4 at D12. Each red or blue dot denotes an individual gene with p ≤ 0.05 and log_2_-fold change>0.25.


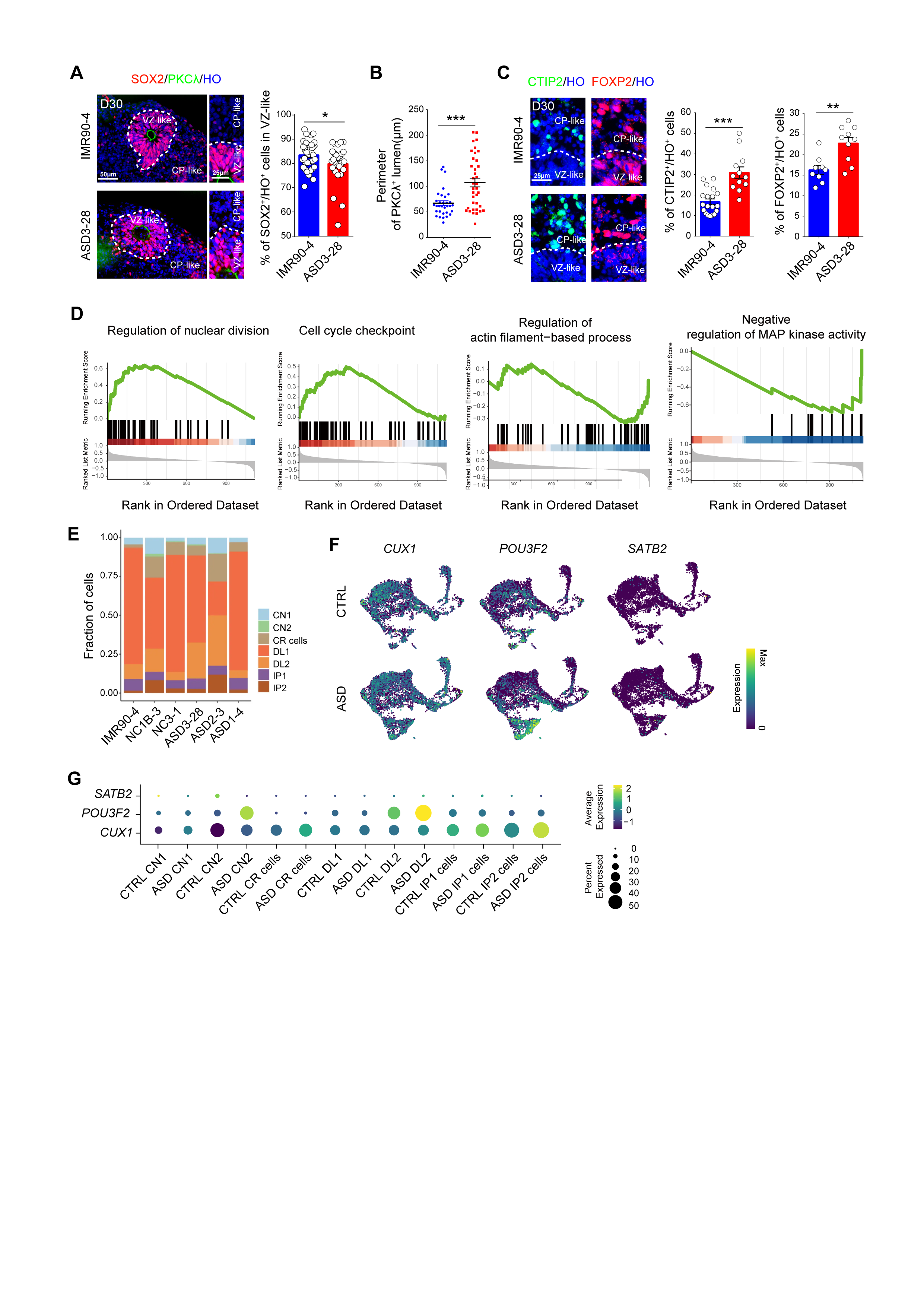


**Figure S3. Premature differentiation of NSCs in ASD organoids at D30**

1. Representative images of immunostaining for SOX2, PKCλ and Hoechst in D30 control and autism organoids (Left). Scale bar: 50μm. Histograms (Right) displaying quantification of the ratio of SOX2 ^+^/HO^+^ cells in VZ-like zone in IMR90-4 and ASD3-28 organoids (IMR90-4: n=37 VZ-like regions, ASD3-28: n=28 VZ-like regions, * p<0.05). VZ-like regions from 3 independent biological replicate experiments were analyzed for each cell line.
2. Dot graphs showing quantification of lumen perimeters defined by PKCλ^+^ staining in IMR904 and ASD 3-28 D30 organoids (IMR90-4: n=32 lumens, ASD3-28: n=37 lumens, *** p<0.001). Organoids from 3 independent biological replicate experiments were analyzed for each cell line.
3. Representative images (Left) and quantification analysis (Right) of D30 IMR904 and ASD3-28 organoids stained by CTIP2 (IMR90-4: n=19 organoids, ASD3-28: n=12 organoids, *** p<0.001) and FOXP2 (IMR90-4: n=9 organoids, ASD3-28: n=10 organoids, ** p<0.01) in the CP-like zone. Scale bar: 50μm. Organoids from 3 independent biological replicate experiments were analyzed for each cell line.
4. Gene set enrichment analysis (GSEA)-enrichment plots of representative gene sets: regulation of nuclear division, cell cycle checkpoint, regulation of actin filament and negative regulation of MAP kinase activity.
5. Chart displaying the comparisons of cell composition in CN cluster between the CRTL and ASD cell lines at D30. CR: Cajal-Retzius cells, DL: deep layer cells, IP: intermediate progenitor.
6. Feature plots showing differential expression of the selected upper layer marker *CUX1*, *POU3F2* and *SATB2* on UMAP plots.
7. Dot plots showing the expression of upper layer cell-related genes (*CUX1, POU3F2* and *SATB2*) in all 7 clusters in CTRL and ASD groups.


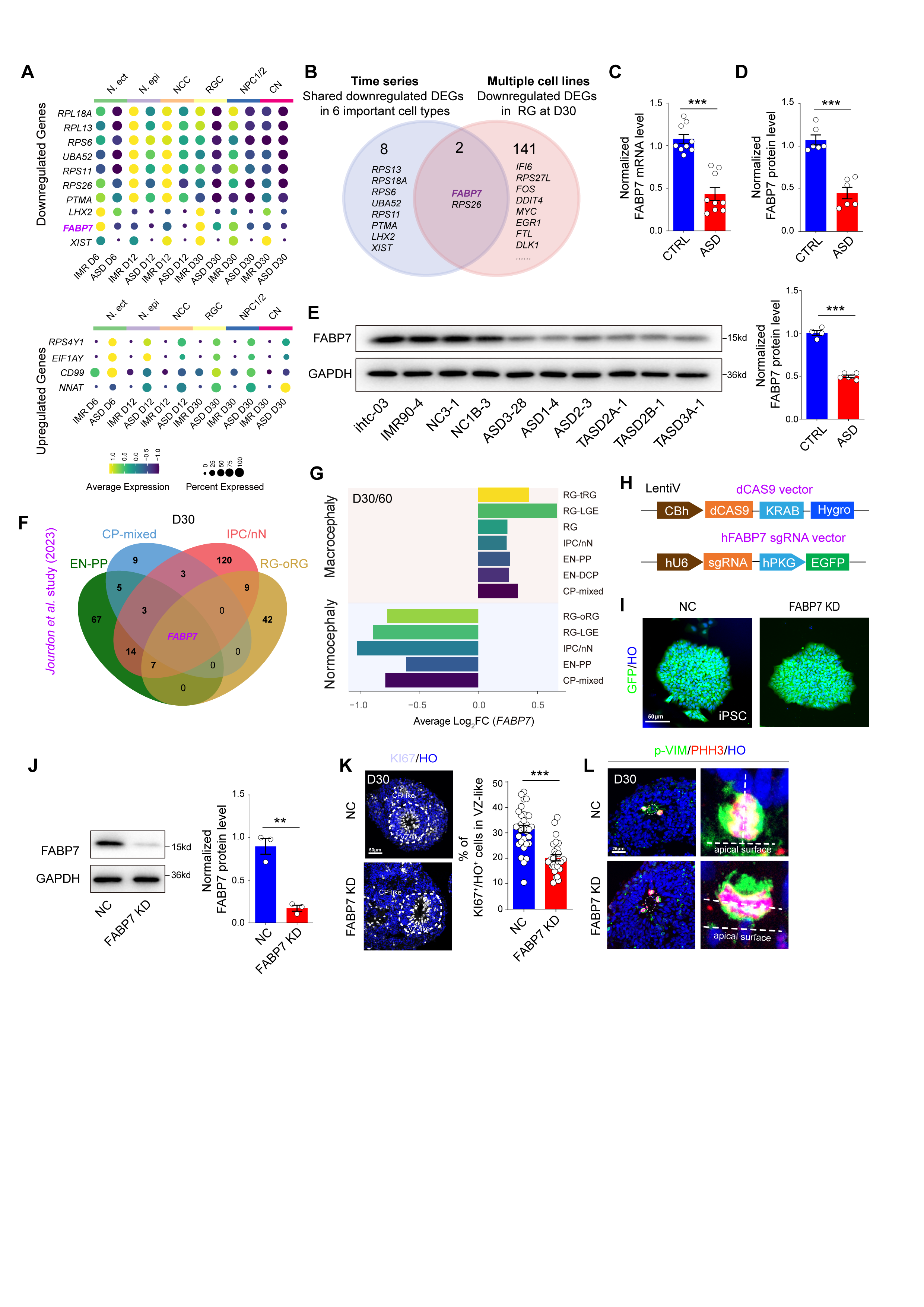


**Figure S4. FABP7 deficiency leads to premature cortical differentiation in ASD organoids**

1. Dot plot showing average and percent expression of ten overlap genes (Top) among ASD downregulated genes and four overlap genes (Bottom) among ASD upregulated genes across 6 cell types.
2. Ven chart showing the shared downregulated DEGs between time series and multiple cell lines single cell RNA-seq data.
3. Normalized *FABP7* mRNA expression levels in D30 CRTL and ASD organoids (IMR90-4: n=3 technical replicates, NC3-1: n=3 technical replicates, NC1B-3: n=3 technical replicates, ASD3-28: n=3 technical replicates, ASD2-3: n=3 technical replicates, ASD1-4: n=3 technical replicates, ASD versus CRTL: *** p<0.001). Organoids (n≥15) from 3 technical replicates were analyzed for each cell line.
4. Relative quantification for the protein level of FABP7 in the CRTL and ASD organoids at D30 (IMR90-4: n=2 independent replicates, NC3-1: n=2 independent replicates, NC1B-3: 2 independent replicates, ASD3-28: n=2 independent replicates, ASD2-3: n=2 independent replicates, ASD1-4: n=2 independent replicates, ASD versus CRTL: *** p<0.001). Organoids (n≥15) from 2 independent biological replicate experiments were analyzed for each cell line.
5. Representative western blots of FABP7 expression (Left) and relative quantification (Right) for the protein level of FABP7 in the CTRL and ASD organoids at D30 (CTRL: n=4 cell lines, ASD: n=6 cell lines, CTRL versus ASD: *** p<0.001). Organoids (n≥15) were analyzed for each cell line.
6. Venn diagram illustrating the intersection of four clusters (CP-mixed, EN-PP, IPC/nN, and RG-oRG) sourced from *Jourdon* et al. study, representing the 30-day condition. The diagram emphasizes the shared elements among different dataset combinations, with particular attention to the central intersection encompassing the only key gene, FABP7. Radial glia- outer radial glia: RG-oRG, intermediate progenitor cells or newborn neurons: IPC/nN, cortical plate mixed neurons: CP-mixed, excitatory neurons included early-born neurons of the preplate: EN-PP.
7. Comparison of *FABP7* expression levels in individuals with macrocephaly and those with normal head circumference over a D30 period, as reported by *Jourdon* et al. The data indicates an increase of *FABP7* expression in the macrocephalic group and a significant decrease in the normal head circumference group. RG expressing truncated RG genes: RG-tRG, dorsal cortical plate ENs: EN-DCP.
8. Schematic of Lentivirus (LentiV) vectors for expressing CRISPRi dual-vectors, dCas9 and h*FABP7* sgRNA vectors.
9. Representative images of GFP in iPSCs in the NC and *FABP7* KD groups. Scale bar: 50μm.
10. Representative western blots of FABP7 expression (Left) and relative quantification (Right) for the protein level of FABP7 in the *FABP7* KD and NC organoids at D30 (NC: n=3 independent replicates, *FABP7* KD: n=3 independent replicates, NC versus *FABP7* KD: ** p<0.01). Organoids (n≥15) from 3 independent biological replicate experiments were analyzed for each cell line.
11. Representative images of immunostaining for KI67 and Hoechst in D30 NC and *FABP7* KD organoids (Left). Scale bar: 50μm. Histograms (Right) displaying quantification of the ratio of KI67 ^+^/HO^+^ cells in VZ-like zone in NC and *FABP7* KD organoids (NC: n=32 VZ-like regions, FABP7 KD: n=27 VZ-like regions, *** p<0.001). VZ-like regions from 3 independent biological replicate experiments were analyzed for each cell line.
12. Representative immunofluorescence images of p-VIM and PHH3 illustrating individual angles of proliferating progenitor cells in the NC and *FABP7* KD D30 organoids. Scale bar: 25 μm.


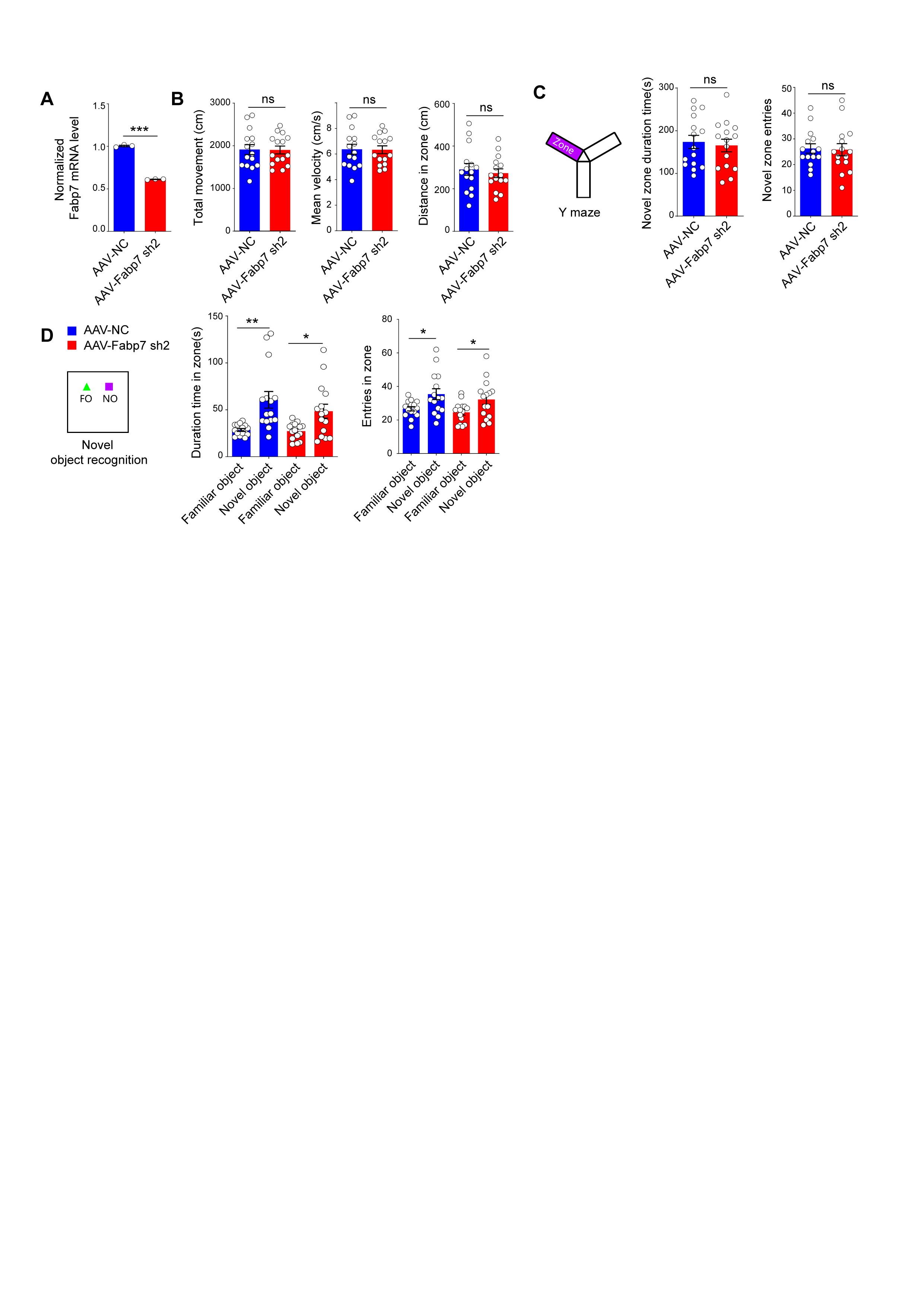


**Figure S5. Behavioral tests of mice with Fabp7 knockdown in the hippocampus**

1. Histograms showing dramatical reduction of the *Fabp7* mRNA levels in AAV-*Fabp7* sh2 groups compared with AAV-NC groups (AAV-NC: n=3 technical replicates, AAV-*Fabp7* sh2: n=3 technical replicates, *** p<0.001).
2. Histograms presenting total movement (AAV-NC: n=15 mice, AAV-*Fabp7* sh2: n=15 mice, ns p=0.95), the mean velocity (AAV-NC: n=15 mice, AAV-*Fabp7* sh2: n=15 mice, ns p=0.94) and distance in zone (AAV-NC: n=15 mice, AAV-*Fabp7* sh2: n=15 mice, ns p=0.62) of the mice in the AAV-NC and AAV-*Fabp7* sh2 groups.
3. Schematic of Y maze test (Left) and histograms (Right) presenting the novel zone duration time (AAV-NC: n=15 mice, AAV-*Fabp7* sh2: n=15 mice, ns p= 0.70) and novel zone entries (AAV-NC: n=15 mice, AAV-*Fabp7* sh2: n=15 mice, ns p= 0.88) of mice in AAV-NC and AAV-*Fabp7* sh2 groups.
4. Schematic of the novel object recognition test (Left) and histograms (Right) displaying the duration (AAV-NC: FO: n=15 mice, NO: n=15 mice; ** p<0.01; AAV-*Fabp7* sh2: FO: n=15 mice, NO: n=15 mice, * p<0.05) and entries (AAV-NC: FO: n=15 mice, NO: n=15 mice, * p<0.05; AAV-*Fabp7* sh2: FO: n=15 mice, NO: n=15 mice, * p<0.05) of mice in the novel object (NO) zone compared with the familiar object (FO) zone (green) in the AAV-NC and AAV- *Fabp7* sh2 groups.


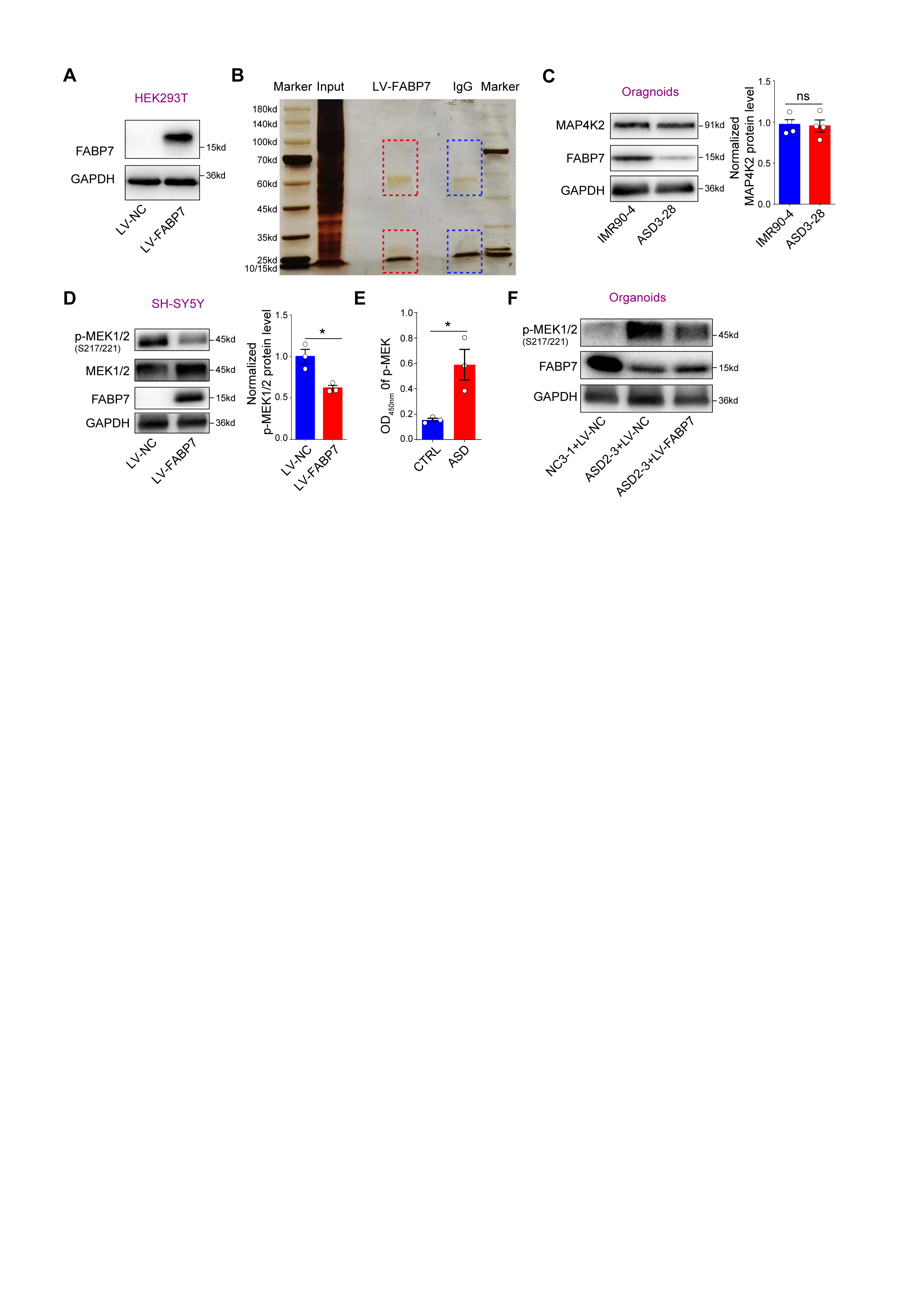


**Figure S6. FABP7 regulates premature neural differentiation through interactions with MAPK signals in ASD organoids**

1. Representative western blots of FABP7 expression in the HEK293T cells after LV-NC and LV-*FABP7* infection.
2. Optical imaging of polyacrylamide gel electrophoresis (PAGE) gel with silver staining after running input, *FABP7* OE and IgG samples. Red and Bule dotted boxes in *FABP7* OE and IgG lanes, respectively, represented the areas were cut for Label-free analysis.
3. Representative western blots of MAP4K2 and FABP7 expression (Left) and relative quantification (Right) in IMR904 and ASD3-28 organoids at D30 (IMR90-4: n=4 independent replicates, ASD3-28: n=4 independent replicates, ns p= 0.8572). Organoids (n≥15) from 4 independent biological replicate experiments were analyzed for each cell line.
4. Representative western blots of p-MEK1/2, MEK1/2 and FABP7 expression in SH-SY5Y cells after infection with control and *FABP7* OE lentivirus (Left). Histograms (Right) showing the relative quantification of p-MEK1/2 in SH-SY5Y cells in the NC and *FABP7* OE groups (NC: n=3 independent replicates, *FABP7* OE: n=3 independent replicates, * p<0.05).
5. Histograms showing the relative OD values of p-MEK in the CRTL and ASD organoids at D30 (IMR90-4: 0.1534, NC3-1:0.1317, NC1B-3: 0.1782, ASD3-28: 0.8032, ASD2-3: 0.384, ASD1-4:0.5818, ASD versus CRTL: * p<0.05; Standard curve: y = 5.6617x - 0.0269, R2=0.9989). Organoids (n≥15) were analyzed for each cell line.
6. Representative western blots of p-MEK1/2 and FABP7 expression in NC3-1+LV-NC, ASD2-3+LV-NC and ASD2-3+LV-FABP7 organoids at D30.


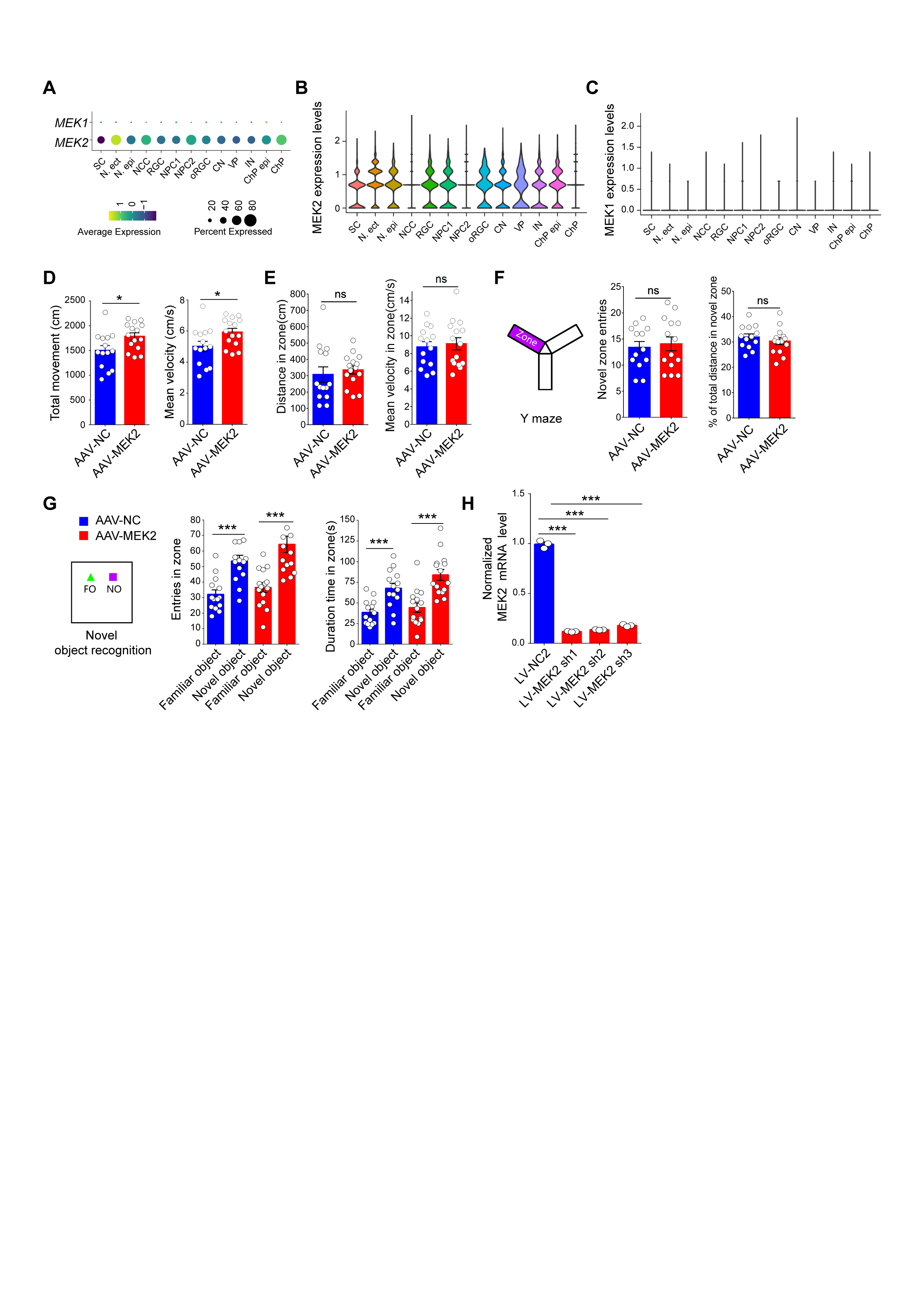


**Figure S7.** **Behavioral tests of mice with MEK2 overexpression in the hippocampus and verification of MEK2 knockdown after virus infection**

1. Dot plots showing the expression of *MEK1* and *MEK2* in all cell types over 6 developmental time points.
2. Violin plots displaying the expression of *MEK2* in all cell types over 6 developmental time points.
3. Violin plots displaying the expression of *MEK1* in all cell types over 6 developmental time points.
4. Schematic of the open field test (Left) and histograms (Right) displaying the total movement (AAV-NC: n=14 mice, AAV-*MEK2*: n=15 mice, * p<0.05) and mean velocity (AAV-NC: n=14 mice, AAV-*MEK2*: n=15 mice, * p<0.05) of mice in AAV-NC and AAV-*MEK2* groups in the open field test.
5. Histograms showeing the distance in zone (AAV-NC: n=14 mice, AAV-*MEK2*: n=15 mice, ns p= 0.60) and mean speed in zone (AAV-NC: n=14 mice, AAV-*MEK2*: n=15 mice, ns p= 0.70) in AAV-NC and AAV-*MEK2* groups in the open field test.
6. Schematic of Y maze test (Left) and histograms (Right) presenting the novel zone duration time (AAV-NC: n=13 mice, AAV-*MEK2*: n=14 mice, ns p= 0.19), novel zone entries (AAV-NC: n=13 mice, AAV-*MEK2*: n=14 mice, ns p= 0.70) and total distance in novel zone (AAV-NC: n=13 mice, AAV-*MEK2*: n=14 mice, ns p= 0.70) of mice in AAV-NC and AAV-*MEK2* groups.
7. Schematic of the novel object recognition test (Left) and histograms (Right) displaying the entries (AAV-NC: FO: n=14 mice, NO: n=14 mice; *** p<0.001; AAV-*MEK2*: FO: n=15 mice, NO: n=15 mice, *** p<0.001) and duration (AAV-NC: FO: n=14 mice, NO: n=14 mice, *** p<0.001; AAV-*MEK2*: FO: n=15 mice, NO: n=15 mice, *** p<0.001) of mice in the novel object (NO) zone compared with the familiar object (FO) zone in the AAV-NC and AAV-*MEK2* groups.
8. Histograms showing dramatical reduction of the mRNA levels in LV-NC2, LV-*MEK2* sh1, LV-*MEK2* sh2 and LV-*MEK2* sh3 groups (LV-NC2: n=3 technical replicates, LV-*MEK2* sh1: n=3 technical replicates, LV-*MEK2* sh2: n=3 technical replicates, LV-*MEK2* sh3: n=3 technical replicates, LV-*MEK2* sh1 versus NC, *** p<0.001. LV-*MEK2* sh2 versus NC, *** p<0.001. LV-*MEK2* sh3 versus NC, *** p<0.001).
